# Evaluating primary health care utilization among lesbian, gay, and bisexual individuals in Canada with a view to inform more equitable health measures

**DOI:** 10.1186/s12889-026-26949-6

**Published:** 2026-03-09

**Authors:** Ivan Marbaniang, Erica E.M. Moodie, Eric Latimer, Joseph Cox

**Affiliations:** 1 Department of Epidemiology, Biostatistics and Occupational Health, McGill University, 2001 McGill College Avenue, suite 1200, Montreal, QC H3A 1G1 Canada; 2 Mental Health and Society Division, Douglas Research Centre, Montreal, QC Canada; 3 Department of Psychiatry, McGill University, Montreal, QC Canada; 4 Direction régionale de santé publique, Centre intégré universitaire de santé et de services sociaux du Centre-Sud-de-l’Île-de-Montréal, Montreal, QC Canada; 5 Clinical Outcomes Research and Evaluation, Research Institute–McGill University Health Centre, Montreal, QC Canada

**Keywords:** Primary Health Care, Health Services Utilization, Sexual Minority Persons, Canada

## Abstract

**Background:**

Canada is advancing several reforms to strengthen primary health care. Although lesbian, gay, and bisexual (LGB) individuals experience a higher burden of some health conditions, they remain largely absent from these reforms, partly because of limited data. We assessed differences in primary care utilization between LGB and heterosexual individuals to address this gap.

**Methods:**

We used data from 91,172 heterosexual and 2,550 LGB participants in the 2015–2016 Canadian Community Health Survey. Guided by Andersen’s Behavioural Model of Health Services Use, we examined the association between primary care physician (PCP) consultation rates and our primary variables of interest: sex and sexuality. Estimates for heterosexual men, heterosexual women, gay and bisexual men (GBM), and lesbian and bisexual women (LBW) were obtained separately. Models were adjusted for additional covariates (predisposing, enabling, and need-based) identified in Andersen’s framework. Poisson regression was used for the main analysis, and results were compared with estimates from double-selection lasso models.

**Results:**

GBM and LBW had higher PCP consultation rates over a 12-month period compared with heterosexual men. For every 100 PCP consultations among heterosexual men, there were an estimated 129 consultations (95% CI 112 to 148) among GBM and 123 consultations (95% CI 110 to 139) among LBW. In contrast, consultation rates for GBM and LBW were comparable with heterosexual women.

**Conclusion:**

Even after adjusting for several covariates, GBM and LBW had higher PCP consultation rates than heterosexual men. These findings suggest that PCP engagement among LGB adults differs from that of heterosexual men. The comparability with heterosexual women requires further investigation. More comprehensive data are needed to verify and clarify these findings to support equitable primary care planning.

**Supplementary Information:**

The online version contains supplementary material available at 10.1186/s12889-026-26949-6.

## Introduction

In Canada, approximately 70% of healthcare is publicly funded, overseen by ten provincial and three territorial health systems [1]. The Canada Health Care Act stipulates that despite this decentralized structure, the provision of non-discriminatory patient-centered care, emphasizing comprehensiveness and continuity, remains a foundational principle of primary health care across the country [2]. Underlying this principle is the premise that every individual, regardless of their social identities (e.g., sexuality, gender, race, socioeconomic status) is entitled to high-quality primary health care.

Over time, however, Canada’s primary care system has struggled to deliver timely, high-quality care to many of its residents [3, 4]. Contributing factors include chronic underinvestment, stagnation in the primary care physician workforce, outdated payment models that burden physicians with administrative work, and an aging population with increasingly complex needs [4, 5]. The 2023 Commonwealth Fund Survey found that Canada ranked last among ten high-income countries for access to a primary care provider, lagging even behind the United States, which lacks universal coverage [6]. Furthermore, an Angus Reid survey found that among those without a family doctor, 38% had been searching for at least a year and 26% had stopped looking altogether [3, 7].

Lesbian, gay, and bisexual (LGB) individuals experience a greater prevalence of certain physical and mental health conditions compared to their heterosexual counterparts. While existing research has primarily focused on disparities on health problems such as sexually transmitted infections (e.g., HIV, syphilis) [8], substance use disorders [9], depression [10], and suicidality [11], emerging evidence indicates higher occurrence of conditions like arthritis [12], diabetes [13], hypertension [14], chronic respiratory illnesses [15, 16], and certain cancers among LGB individuals [17]. Our understanding of the underlying mechanisms driving these disparities is also advancing. Traditionally, *minority stress theory* has attributed LGB health disparities to maladaptive psychological and biological pathways resulting from the heightened discrimination-related stress of being a sexual minority [18]. This theory positions discrimination and stigma as primary drivers of stress-related dysregulation. On the other hand, the concept of *insufficient social safety* emphasizes the absence of reliable social connection, inclusion, and protection, which produces chronic threat-vigilance even in the absence of overt discrimination [19]. It highlights how lacking dependable social resources, rather than exposure to discrimination alone, sustains physiological and psychological strain. *Fundamental cause theory* further underscores how the persistent non-addressal of systemic factors such as stigma generates new and evolving mechanisms that reproduce health inequities over time [20]. Together, these frameworks complement and expand our understanding of the structural and relational determinants shaping LGB health disparities.

Given that many health disparities affecting LGB individuals are amenable to primary (e.g., pre-exposure prophylaxis for HIV, vaccination against HPV) and secondary (e.g., screening for depressive symptoms, substance use disorders, and cancers) prevention interventions, the importance of primary care in mitigating these inequities cannot be overstated. Moreover, primary care, with its emphasis on comprehensive care, extends beyond curative measures (including prevention, health education, coordination with social services etc.) [2]. This broader approach may uniquely position primary care providers to respond holistically to the diverse health needs of LGB patients, acknowledging the impact of sexuality on health without making it the sole determinant of health care needs [21].

Despite the potential of primary care to address LGB health disparities, LGB individuals remain largely overlooked in primary care research in Canada. OurCare, the largest pan-Canadian survey to date with broad community involvement, did not collect data on sexual identity and included only a single community-based roundtable for sexual and gender diverse individuals [22]. Amid ongoing discussions about restructuring the country’s primary care system, including measures such as strengthening team-based practice and expanding the clinical role of allied health professionals (e.g., pharmacists) [4, 5], the absence of sexual identity data makes it difficult to plan evidence-based LGB-specific interventions. This is particularly concerning because primary care settings are well positioned to integrate approaches informed by the growing understanding of the social and structural drivers of LGB health inequities [23, 24].

Health care utilization is influenced by a complex interplay of behavioural processes and structural factors, encompassing the ability to recognize the need for care, access care, and actively engage in care [25]. Understanding primary care utilization levels among LGB individuals can clarify how engagement differs across groups and inform more equitable resource planning. However, few studies in Canada have examined primary care utilization among LGB individuals, and most quantitative research dichotomizes use as any use versus non-use, limiting interpretation of engagement in care [26, 27].

In this manuscript, we assess and compare the rates of primary health care consultations among gay and bisexual men, lesbian and bisexual women, heterosexual women and heterosexual men.

## Methods

### Study population

**Fig. 1:**
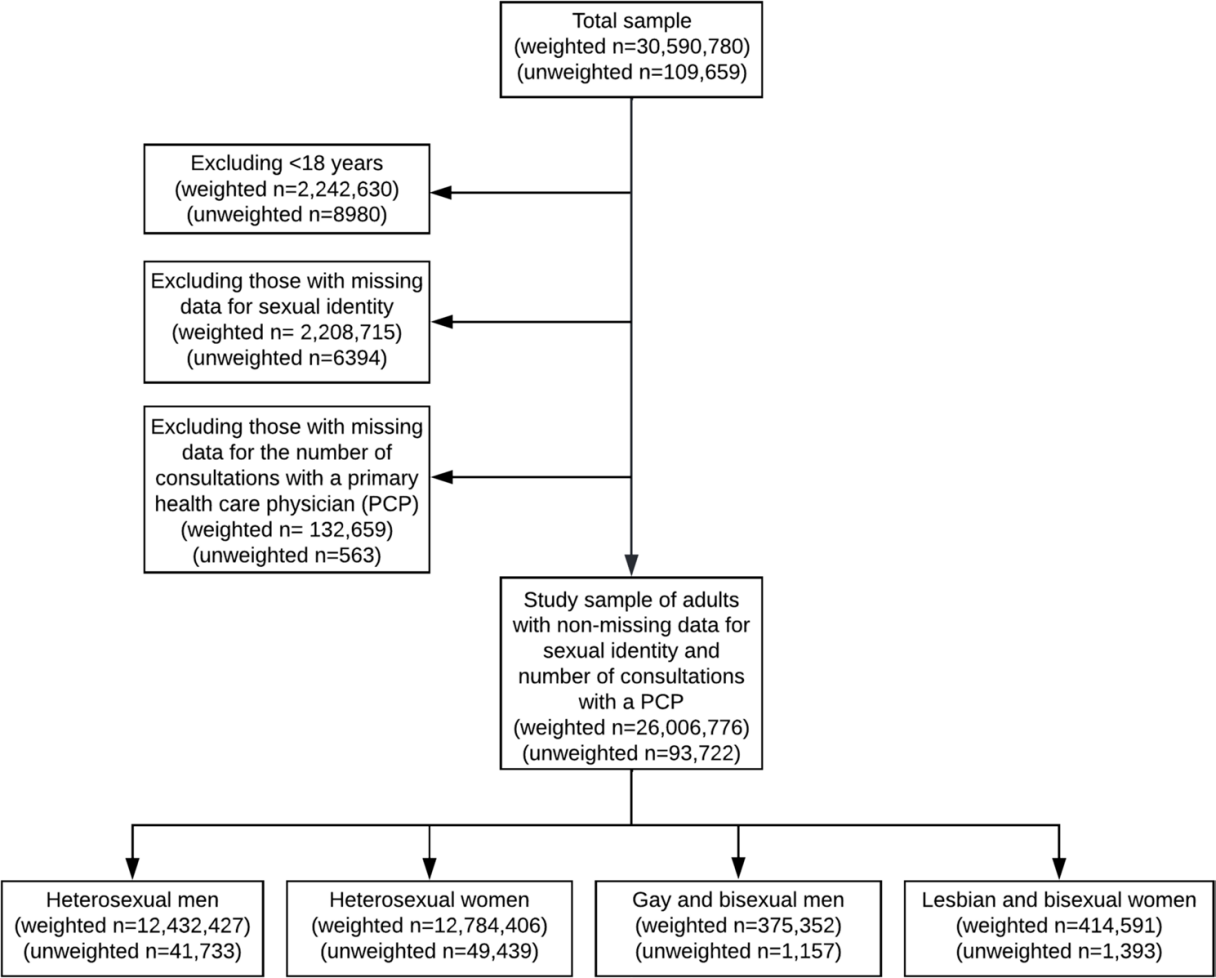
Flowchart showing the study population (unweighted and weighted) and excluded participants, using data from the 2015–2016 Canadian Community Health Survey

We used data from the Public Use Microdata File (PUMF) of the 2015–2016 Canadian Community Health Survey (CCHS). Details on CCHS can be found elsewhere [28]. Briefly, the CCHS is a nationwide complex cross-sectional survey representing approximately 97% of non-institutionalized civilians living in Canada’s ten provinces and three territories. Participants are selected using multistage random sampling and complete health-related questionnaires through computer-assisted interviews. For our analyses, we included respondents ≥ 18 years of age with complete data for sexual identity and number of consultations with a family doctor or general practitioner in the previous 12 months (Fig. 1). In the remainder of the manuscript, we will use primary care physician (PCP) to mean family doctor or general practitioner.

### Theoretical model and study definitions

We used Andersen’s Behavioural Model of Health Services Use (BMHSU) to guide our analyses [25]. BMHSU identifies **predisposing** socio-demographic, **enabling** logistical and economic, and **need-based** (self-reported and evaluated) factors that affect health care use. While sexual identity was not included in the original model, a later extension of BMHSU for vulnerable populations proposed it to be a predisposing factor [29].

The **outcome** was the self-reported number of consultations with a PCP in the previous 12 months.

The **explanatory variable of interest** was self-identified sexual identity (two groups: heterosexual, gay or lesbian & bisexual). Gay and bisexual men were grouped together (GBM), and lesbian and bisexual women were grouped together (LBW). To allow for the possibility of different PCP consultation rates between men and women, we utilized sex as a potential effect modifier (i.e., an interaction with the explanatory variable), thus obtaining estimates for heterosexual women, GBM and LBW versus heterosexual men (reference group). As the 2015–2016 CCHS did not collect data on gender identity, the variable “sex” represents sex as reported rather than sex assigned at birth.

Covariates included other factors described in BMHSU and available in the CCHS. Predisposing covariates included age, marital and studentship statuses, education, racial identity, being born in Canada, heavy drinking, and sense of belonging to the local community (as a proxy for social networks specified in BMHSU). Enabling covariates included geographical regions of Canada as classified by Statistics Canada, annual household income, having a regular health care provider, insurance coverage for medication, and living arrangement (as a proxy for social support in BMHSU). Need-based covariates included both self-perceived measures (general health, mental health, and life stress) and professionally evaluated measures (number of chronic physical health conditions and having a mood or anxiety disorder). We also included emergency room visits in the previous 12 months as a covariate to account for health care-seeking behaviour that may otherwise be reflected in PCP consultation counts [29]. These 19 covariates were categorized as presented in Table 1.

**Table 1:** Weighted estimates from the 2015-2016 Canadian Community Health Survey for factors in the Andersen’s Behavioural Model of Health Services Use (BMHSU), by sex and sexual identity

	**Total** (n= 26,006,776) % (95% CI)	** Men **(n= 12,807, 779)		** Women **(n=13,198, 997)	
		Heterosexual (n=12,434,427) % (95% CI)	Gay and bisexual (n=375,352) % (95% CI)	Heterosexual (n=12,784,406) % (95% CI)	Lesbian and bisexual (n=414,591) % (95% CI)
*Predisposing factors *					
**Age (years)**					
18 – 29	19.7 (19.4, 20.1)	20.1 (19.5, 20.6)	31.2 (26.1, 36.7)	18.4 (17.9, 18.8)	42.8 (38.3, 47.4)
30 – 39	17.9 (17.4, 18.3)	18.0 (17.4, 18.6)	16.8 (13.5, 20.8)	17.7 (17.1, 18.3)	20.1 (16.7, 23.9)
40 – 49	17.3 (17.0, 17.6)	17.6 (17.1, 18.0)	14.7 (11.4, 18.7)	17.3 (16.9, 17.7)	12.3 (9.5, 15.7)
50 – 59	18.7 (18.4, 19.0)	19.1 (18.7, 19.6)	18.6 (15.1, 22.8)	18.5 (18.1, 18.9)	12.6 (10.1, 15.6)
60 – 69	15.3 (15.0, 15.6)	15.2 (14.8, 15.7)	11.4 (8.4, 15.3)	15.7 (15.3, 16.2)	9.3 (6.0, 14.0)
≥70	11.1 (10.9, 11.3)	10.0 (9.7, 10.3)	7.3 (5.3, 9.9)	12.5 (12.2, 12.8)	2.9 (2.2, 3.9)
**Education**					
< Secondary education	11.1 (10.8, 11.4)	11.1 (10.7, 11.6)	5.4 (4.0, 7.2)	11.2 (10.8, 11.6)	13.0 (9.4, 17.5)
Secondary and < post secondary education	22.8 (22.4, 23.3)	23.2 (22.5, 23.9)	23.4 (19.0, 28.4)	22.3 (21.7, 22.9)	26.0 (22.5, 29.9)
≥ Post-secondary education	64.7 (64.1, 65.2)	64.3 (63.5, 65.1)	69.6 (64.5, 74.2)	65.1 (64.4, 65.8)	60.3 (55.6, 64.9)
Missing	1.3 (1.2, 1.5)	1.3 (1.2, 1.6)	1.6 (0.8, 3.3)	1.4 (1.2, 1.6)	0.7 (0.3, 1.7)
**Student**					
Yes	9.6 (9.2, 10.0)	8.5 (8.1, 9.0)	13.8 (10.3, 18.4)	10.0 (9.6, 10.7)	20.6 (16.8, 25.0)
No	89.7 (89.3, 90.1)	90.6 (90.1, 91.1)	85.5 (80.9, 89.1)	89.3 (88.8, 89.8)	78.3 (73.9, 82.2)
**Racial identity** ^a^					
White	75.4 (74.9, 75.9)	75.2 (74.4, 75.9)	79.2 (74.8, 82.9)	75.5 (74.8, 76.2)	78.1 (73.8, 81.8)
Racialized	20.3 (19.8, 20.8)	20.6 (19.8, 21.4)	17.1 (13.5, 21.3)	20.3 (19.6, 21.0)	13.7 (10.6, 17.7)
Missing	4.2 (4.0, 4.4)	4.2 (3.9, 4.6)	3.7 (2.7, 5.2)	4.1 (3.8, 4.4)	8.2 (6.3, 10.6)
**Canadian born**					
Yes	74.1 (73.5, 74.6)	73.7 (72.9, 74.5)	78.3 (73.8, 82.2)	73.9 (73.2, 74.6)	84.7 (80.6, 88.1)
No	25.3 (24.7, 25.8)	25.6 (24.8, 26.4)	20.2 (16.4, 24.7)	25.5 (24.8, 26.2)	14.4 (11.1, 18.5)
**Marital status**					
Single	24.6 (24.1, 25.1)	26.0 (25.3, 26.7)	55.2 (50.1, 60.2)	21.5 (21.0, 22.1)	51.0 (46.3, 55.8)
Married or common-law	62.4 (61.7, 63.2)	65.5 (64.5, 66.4)	36.4 (31.7, 41.4)	61.0 (60.2, 61,9)	38.8 (34.2, 43.6)
Widowed, separated, divorced	12.7 (12.2, 13.2)	8.3 (7.8, 8.8)	8.2 (6.5, 10.5)	17.2 (16.6, 17.8)	10.1 (7.9, 12.8)
**Sense of belonging to local community**					
Very strong	17.6 (17.1, 18.0)	17.6 (17.0, 18.2)	15.2 (12.0, 19.2)	17.9 (17.3, 18.5)	9.7 (7.5, 12.3)
Somewhat strong	48.9 (48.3, 49.4)	48.5 (47.7, 49.3)	41.0 (35.9, 46.3)	49.5 (48.8, 50.3)	46.2 (41.5, 51.0)
Somewhat weak to very weak	32.4 (31.8, 32.9)	32.7 (31.9, 33.5)	43.2 (38.0, 48.7)	31.3 (30.6, 32.1)	42.5 (37.8, 47.4)
Missing	1.2 (1.1, 1.3)	1.2 (1.0, 1.4)	<1%	1.2 (1.1, 1.4)	1.6 (0.7, 3.8)
*Enabling factors *					
**Geographical regions** ^b^					
Atlantic	6.8 (6.7, 6.9)	6.7 (6.6, 6.8)	6.0 (4.6, 7.8)	7.0 (6.9, 7.0)	7.2 (5.8, 9.0)
Quebec	23.6 (23.5, 23.8)	23.5 (23.3, 23.7)	32.2 (28.0, 36.7)	23.5 (23.3, 23.8)	23.7 (20.0, 27.9)
Ontario	37.9 (37.7, 38.1)	37.5 (37.1, 37.8)	37.5 (32.4, 42.9)	38.3 (38.0, 38.6)	39.6 (35.0, 44.5)
Prairies	18.1 (18.0, 18.2)	18.8 (18.7, 19.0)	11.2 (9.0, 13.8)	17.7 (17.5, 17.9)	15.7 (13.1, 18.6)
British Columbia	13.2 (13.1, 13.3)	13.2 (13.0, 13.4)	12.8 (10.1, 16.1)	13.2 (13.1, 13.4)	13.4 (11.0, 16.3)
Territories	0.31 (0.30, 0.31)	0.32 (0.31, 0.32)	0.3 (0.2, 0.4)	0.3 (0.3, 0.3)	0.3 (0.2, 0.4)
**Annual household income**					
< $40,000	21.1 (20.5, 21.7)	18.0 (17.3, 18.7)	23.3 (19.7, 27.5)	23.7 (22.9, 24.5)	30.2 (25.9, 34.8)
$40,000 - $79,999	29.2 (28.7, 29.7)	28.6 (27.9, 29.4)	31.2 (26.5, 36.4)	29.7 (29.0, 30.4)	31.2 (27.2, 35.6)
≥$80,000	49.6 (48.9, 50.4)	53.3 (52.4, 54.3)	45.4 (40.0, 50.8)	46.5 (45.7, 47.4)	38.5 (33.9, 43.4)
**Regular health care provider** ^c^					
Yes	83.1 (82.7, 83.5)	78.8 (78.1, 79.5)	77.5 (73.3, 81.2)	87.6 (87.2, 88.1)	78.6 (73.8, 82.8)
No	16.7 (16.3, 17.2)	21.0 (20.3, 21.7)	22.4 (18.8, 26.6)	12.3 (11.8, 12.7)	21.4 (17.2, 26.2)
**Insurance covers all or part of medication cost** ^d^					
Yes	79.0 (78.5, 79.5)	78.4 (77.7, 79.1)	75.3 (69.9, 79.9)	79.8 (79.2, 80.4)	76.4 (72.2, 80.0)
No	20.1 (19.6, 20.5)	20.5 (19.8, 21.2)	22.4 (18.0, 27.6)	19.5 (18.8, 20.1)	22.9 (19.3, 27.0)
**Living arrangement** ^e^					
Living alone	15.6 (14.7, 16.6)	14.1 (13.0, 15.2)	29.0 (25.0, 33.3)	16.6 (15.7, 17.6)	18.3 (15.5, 21.4)
Living with others	83.9 (82.9, 84.8)	85.4 (84.3, 86.4)	70.6 (66.2, 74.6)	82.9 (81.9, 93.8)	81.4 (78.3, 84.2)
*Need factors -perceived and evaluated*					
**Self-perceived general health**					
Excellent or very good	61.9 (61.4, 62.4)	62.7 (61.9, 63.5)	63.9 (59.1, 68.4)	61.4 (60.8, 62.1)	49.6 (44.9, 54.2)
Good	27.7 (27.2, 28.2)	27.5 (26.8, 28.2)	25.6 (21.5, 30.2)	27.7 (27.0, 28.4)	35.8 (31.4, 40.4)
Fair or poor	10.3 (10.0, 10.6)	9.7 (9.2, 10.2)	10.5 (8.2, 13.4)	10.7 (10.3, 11.2)	14.6 (11.1, 19.0)
**Self-perceived life stress**					
Not very or not at all stressful	36.0 (35.5, 36.5)	38.2 (37.4, 38.9)	32.8 (28.3, 37.7)	34.4 (33.7, 35.1)	23.4 (19.9, 27.4)
A bit stressful	41.7 (41.2, 42.5)	41.4 (40.6, 42.2)	40.8 (35.9, 46.0)	42.0 (41.3, 42.7)	43.5 (39.1, 48.0)
Quite a bit or extremely stressful	21.9 (21.4, 22.4)	20.1 (19.4, 20.7)	26.1 (21.8, 31.0)	23.2 (22.6, 23.9)	32.7 (28.6, 37.0)
**Self-perceived mental health**					
Excellent or very good	71.6 (71.0, 72.1)	73.7 (73.0, 74.4)	65.1 (60.3, 69.7)	70.4 (69.7, 71.1)	47.6 (43.3, 52.0)
Good	21.9 (21.4, 22.4)	20.6 (20.0, 21.3)	22.8 (19.0, 27.1)	22.8 (22.1, 23.4)	31.9 (27.4, 36.7)
Fair or poor	6.4 (6.1, 6.7)	5.4 (5.1, 5.8)	12.0 (9.3, 15.4)	6.7 (6.3, 7.1)	20.4 (16.9, 24.4)
**Chronic physical health conditions** ^f^					
None	54.2 (53.7, 54.7)	54.8 (54.0, 55.6)	61.1 (55.9, 66.1)	53.4 (52.7, 54.1)	55.0 (50.4, 59.5)
One	23.2 (22.8, 23.7)	23.0 (22.3, 23.7)	22.0 (17.8, 26.8)	23.6 (23.0, 24.2)	20.9 (17.6, 24.6)
Two	11.8 (11.5, 12.1)	11.7 (11.2, 12.2)	9.4 (7.1, 12.3)	12.0 (11.5, 12.4)	11.3 (8.8, 14.2)
≥Three	10.7 (10.5, 11.0)	10.5 (10.1, 10.9)	7.5 (5.7, 9.8)	11.0 (10.6, 11.4)	12.9 (9.3, 17.5)
**Mood or anxiety disorder** ^g^					
Yes	12.3 (11.9, 12.7)	8.5 (8.1, 9.0)	19.7 (16.2, 23.6)	14.8 (14.4, 15.4)	40.0 (35.3, 44.6)
No	87.5 (87.1, 87.8)	91.3 (90.8, 91.7)	80.1 (76.2, 83.5)	84.9 (84.4, 85.4)	59.8 (55.1, 64.4)
*Health Behaviors*					
**Heavy drinker** ^h^					
Yes	21.0 (20.5, 21.4)	26.1 (25.5, 26.8)	31.0 (26.4, 36.1)	15.4 (14.9, 16.0)	26.8 (22.9, 31.2)
No	78.4 (78.0, 78.9)	73.1 (72.4, 73.8)	68.8 (63.8, 73.5)	84.1 (83.5, 84.6)	72.6 (68.2, 76.6)
Any emergency room visit in the previous 12 months					
Yes	24.1 (23.7, 24.6)	23.0 (22.4, 23.7)	20.1 (16.9, 23.8)	25.1 (24.5, 25.7)	31.9 (28.1, 35.9)
No	75.7 (75.3, 76.2)	76.9 (76.2, 77.5)	79.9 (76.2, 83.1)	74.8 (74.2, 75.4)	67.9 (63.8, 71.7)
**Mean number of emergency room visits in the previous 12 months**	0.44 (0.43, 0.45)	0.40 (0.38, 0.42)	0.37 (0.29, 0.44)	0.47 (0.46, 0.49)	0.79 (0.63, 0.95)
**Two or more (≥2) emergency room visits in the previous 12 months**					
Yes	8.9 (8.6, 9.2)	7.8 (7.4, 8.2)	8.9 (6.6, 11.8)	9.7 (9.3, 10.2)	13.8 (11.3, 16.6)
No	91.0 (90.7, 91.3)	92.1 (91.6, 92.5)	91.1 (88.2, 93.4)	90.1 (89.7, 90.5)	86.0 (83.2, 88.5)
**Any consultation with a primary care physician (PCP) in the previous 12 months**					
Yes	69.0 (68.5, 69.5)	62.7 (61.9, 63.4)	64.0 (58.6, 69.0)	75.2 (74.5, 75.9)	74.8 (69.9, 79.2)
No	31.0 (30.5, 31.5)	37.3 (36.6, 38.1)	36.0 (31.0, 41.4)	24.8 (24.1, 25.5)	25.2 (20.8, 30.1)
**Mean number of consultations with a PCP in the previous 12 months**	2.29 (2.26, 2.33)	1.91 (1.87, 1.96)	2.39 (2.04, 2.73)	2.63 (2.58, 2.69)	3.02 (2.64, 3.40)
**Two or more (≥2) consultations with a PCP in the previous 12 months**					
Yes	47.4 (46.8, 47.9)	41.1 (40.3, 41.9)	46.8 (41.7, 52.0)	53.2 (52.5, 54.0)	55.1 (50.4, 59.7)
No	52.6 (52.1, 53.2)	58.9 (58.1, 59.7)	53.2 (48.0, 58.3)	46.8 (46.0, 47.5)	44.9 (40.3, 49.6)

All 95% Confidence Intervals are derived using 1000 bootstrap replicate weights provided by Statistics Canada For variables where a missing category has not been provided, missingness is <1%

^a^The racialized group includes Indigenous Peoples not living on reserves and those belonging to ethnically minority groups (visible minority), defined as per the Canadian federal Employment Equity Act. The term racialized is used as defined in the Government of Canada’s IRCC Anti-Racism Strategy 2.0 (2021-2024) glossary. Indigenous peoples living on reserves are excluded in the CCHS

^b^Geographical regions classified according to the Standard Geographical Classification of Statistics Canada:  Atlantic includes the provinces of Newfoundland and Labrador, Prince Edward Island, Nova Scotia & New Brunswick; Prairies include Manitoba, Saskatchewan & Alberta; Territories include Yukon, Northwest Territories and Nunavut

^c^Regular health care provider included family doctor/general practitioner (97%), medical specialist (1%), nurse practitioner (1%) and other (1%)

^d^Insurance for medications is not publicly funded nationally in Canada, but provinces and territories may have regional plans. For those that have insurance that covers all or part of medication costs: government-sponsored plan (21%), employer-sponsored benefit plan (63%), association (union, trade, student)-sponsored plan (5%), private plan (5%), any combination of the previous four plans (6%)

^e^Living with others included unattached individuals living with others, or individuals living with their spouses/partners and/or children

^f^Chronic physical conditions include living with any of the following: asthma, chronic obstructive pulmonary disease (COPD), arthritis, hypertension, hypercholesterolemia, heart disease, sequalae of stroke, diabetes, cancer, sleep apnea, scoliosis, or fibromyalgia

^g^Mood or anxiety disorder diagnosed by a health professional and lasted/expected to last≥6 months

^h^Heavy drinking is defined as having five or more drinks for male and four or more for female, on one occasion, at least once a month in the previous year, according to Statistics Canada, ‘no’ also includes teetotalers.

Note that, given our study objective, we are primarily interested in interpreting the associations for the explanatory variable of interest and its interaction with sex. The associations with other covariates are not interpreted separately; however, their inclusion in the regression model allows us to estimate the associations for the explanatory variable more clearly, by conditioning (i.e., holding them constant) on other factors posited to be associated with health care use in the BMHSU.

### Statistical analyses

All variables were described using means and proportions with 95% Confidence Intervals (CI). We estimated the association between the outcome and explanatory variable adjusted for covariates using Poisson models, obtaining rate ratios (RRs). All means, proportions, and RRs were weighted using survey weights and 95% CIs obtained using 1000 bootstrap replicate weights available in the CCHS. Effect modification analysis was structured according to the method recommended by Knol and VanderWeele [30], which uses a single reference group, as opposed to traditional stratified analyses that use multiple reference groups. This method allows us to assess the comparability of findings across several categories (e.g., heterosexual men versus heterosexual women, heterosexual women versus LBW, heterosexual women versus GBM, GBM versus LBW, etc.) and gauge the direction of associations for these cross-comparisons (i.e., higher or lower than the reference group), using a single model.

RRs for the association between the outcome and the explanatory variable were obtained using two approaches. First, the *full theoretical model* was used, which aligned with conventional approaches in literature and adjusted for all 19 covariates [31, 32]. Estimates from this model are presented as primary findings. Secondly, we used *double-selection lasso* (least absolute shrinkage and selection operator) regression, which employs a data-dependent selection of covariates followed by a standard Poisson model as proposed by Belloni et al. [33]. For this method, categorical covariates with more than two levels are specified as dummy variables, thus, each categorical level is treated as a separate binary covariate [34]. Hence, the total number of *adjustment factors* increased to 46 (though the number of covariates remained the same, i.e., 19). Using lasso regression for a Poisson model, adjustment factors that predict the outcome and those that predict the explanatory variable are identified in two separate steps (thus, double selection). Then, a standard Poisson model is fit to estimate the association between the outcome and explanatory variable adjusted for the union of variables selected by double-selection lasso. Adjustment factors that predict neither the outcome nor explanatory variable are excluded.

Double-selection lasso has been found to outperform traditional p-value-based selections and stepwise regression in simulation studies [35]. Additionally, while retaining the feature of being able to identify important predictors (and exclude unimportant ones), the double-selection lasso also allows for the determination of CIs and produces estimates less biased to the null: limitations of traditional lasso [35, 36].

We operationalized the double-selection lasso regression in two separate models. First, the pool of predictors was limited to the 46 adjustment factors. Second, recognizing the limitation of assuming linear (one-to-one) relationships between factors in the BMHSU, done conventionally, we included 6 interactions to the pool of predictors (for a total of 96 adjustment factors), based on a review of existing literature. These were interactions between age and number of chronic physical health conditions; racial identity and income; geographic region and having a regular health care provider; having a mood or anxiety disorder and heavy drinking; being born in Canada and sense of belonging to the local community; and having a regular health care provider and insurance coverage for medication.

More details on the double-selection lasso and sources for the interactions selected are provided in the supplementary section.

### Sensitivity analyses

We performed several sensitivity analyses to assess the robustness of our estimates to modelling assumptions. First, 31% of the participants reported zero consultations. We hypothesized that this could be due to two separate processes resulting in excessive zeros: one in which participants did not consult a PCP, another where certain participants did not have a regular PCP and therefore reported zero consults. Accounting for the two processes could lead to different inferences. Therefore, we compared estimates of the full theoretical model fit via a Poisson regression with those using a zero-inflated Poisson model, adjusting for the same covariates. Second, we extended the definition of our explanatory variable for LGB to include participants that may self-identify as heterosexual but reported having had same-sex sexual relationships in the previous year, therefore accounting for both sexual identity and sexual behaviour. We fit a model with this extended definition to determine whether estimates were different from those of the full theoretical model. Third, since we excluded about 8% of participants with missing sexual identity data from the study population, we conducted an ‘extreme case’ imputation assuming in two separate models that those with missing data were either all heterosexual or all LGB. This allowed us to assess the impact of excluding those individuals who did not report sexual identify on the full theoretical model estimates. Lastly, we conducted two further analyses to assess the impact of omitting individuals with missing data from the primary analysis; although each covariate exhibited low missingness, when used together, the analytical sample size was reduced by 12%. In the first of these analyses, we compared estimates from the main model with those obtained after multiply imputing missing covariates values using a chained equations approach. In a second analysis, acknowledging that some GBM may consult a PCP more often for HIV tests, and that HIV testing may generally serve as a proxy for sexually transmitted and bloodborne infections (STBBI) screening and care, we introduced an adjustment variable not considered in the main model: HIV testing in the previous 12 months (yes/no). HIV testing in the previous 12 months exhibited a high degree of missingness (34%); in this final analysis, this variable was also multiply imputed prior to fitting the regression.

We also conducted four post-hoc analyses. First, we evaluated associations separately for individuals with and without chronic physical health conditions, to assess if the primary findings varied by sex and sexual identity across physical health statuses. Second, we examined whether the primary findings held among individuals more consistently engaged in care by restricting the study population to those with at least 1, 2, 3, or 4 PCP consultations. Third, we evaluated associations excluding women on folic acid supplements (as a proxy for pregnancy), to assess whether the findings for women were influenced by possible antenatal consultations. Fourth, we qualitatively examined covariate distributions by number of PCP consultations for heterosexual women, GBM, and LBW to contextualize how profiles of predisposing, enabling, and need-based factors vary across consultation frequencies for these groups.

All analyses were performed in Stata 18.0.

### Ethics approval

The dataset used is publicly available and contains no personal identifiers. Therefore, no ethical review or separate consent procedures were required, in accordance with the Government of Canada’s Tri-Council Policy Statement. The data collection and curation procedures for the CCHS dataset are overseen by Statistics Canada, which ensures that all processes comply with the Declaration of Helsinki.

## Results

Our study population included 93,722 participants representing 26,006,776 people. There were 1,157 participants who identified as GBM representing 375,352 men (2.9% of all men [95% CI 2.6–3.3%]); and 1,393 participants who identified as LBW representing 414,591 women (3.1% of all women, [2.9–3.4%]) (Fig. 1).

Overall, participants were approximately equally distributed across age groups, with the largest difference between age distributions being between those 18–29 (constituting 19.7% [95% CI 19.4–20.1%] of the study population) and ≥ 70 years of age (11.9% [10.9–11.3%]). Most participants identified as white (75.4% [74.9–75.9%]) and lived in Ontario (37.9% [37.7–38.1%]). 83% [82.7–83.5%] of the participants had a regular health care provider and 79% [78.5–79.5%] had insurance that covered all or part of their medication costs. About 69% [68.5–69.5%] of the participants had consulted a PCP in the previous year (Table 1).

Compared with their heterosexual counterparts, more LGB participants reported a ‘somewhat weak to very weak’ sense of belonging to their local communities (GBM: 43.2% [95% CI 38.0-48.7%] vs. heterosexual men: 32.7% [31.9–33.5%]; LBW: 42.5% [37.8–47.4%] vs. heterosexual women: 31.3% [30.6–32.1%]). A higher proportion of LGB participants self-reported ‘fair or poor’ mental health (GBM: 12.0% [9.3–15.4%] vs. heterosexual men: 5.4% [5.1–5.8%]; LBW: 20.4% [16.9–24.4%] vs. heterosexual women: 6.7% [6.3–7.1%]), and were also more likely to be living with a mood or anxiety disorder (GBM: 19.7% [16.2–23.6%] vs. heterosexual men 8.5% [8.1-9.0%]; LBW: 40.0% [35.3–44.6%] vs. heterosexual women: 14.8% [14.4–15.4%]). More GBM (61.1% [55.9–66.1%]) reported not living with a chronic physical health condition than heterosexual men (54.8% [54.0-55.6%]), heterosexual women (53.4% [52.7–54.1%]) and LBW (55.0% [50.4–59.5%]). LBW were more likely to have had two or more emergency room visits in the previous 12 months (13.8% [11.3–16.6%]) relative to heterosexual women (9.7% [9.3–10.2%]), GBM (8.9% [6.6–11.8%]) and heterosexual men (7.8% [7.4–8.2%]) (Table 1).

**Fig. 2:**
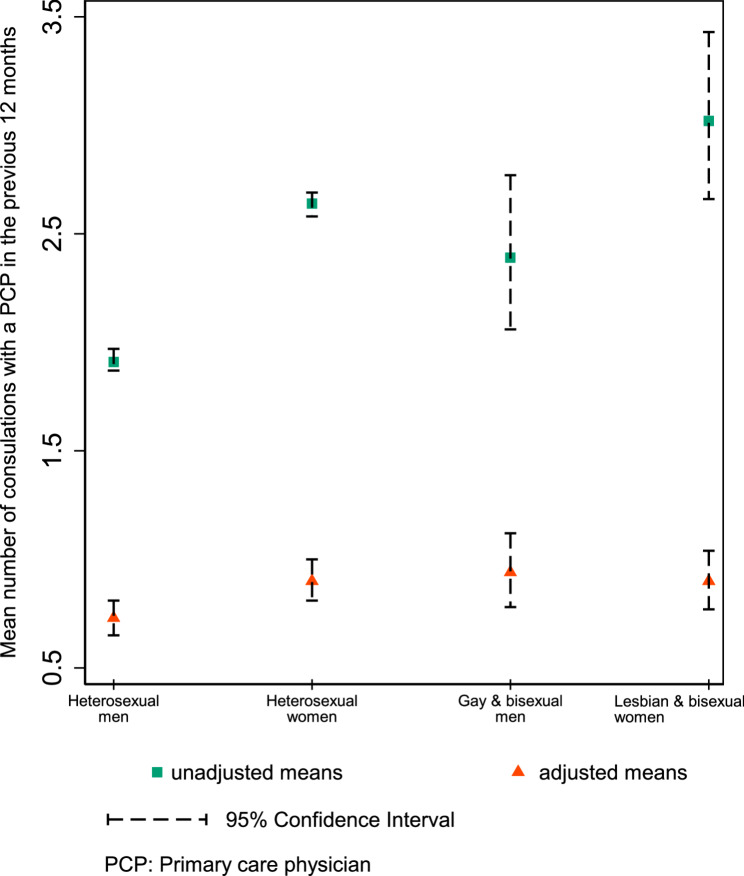
Unadjusted and adjusted (for 19 covariates in Andersen’s Behavioural Model of Health Services Use listed in Table 2) modelled mean number of consultations with a primary care physician in the previous 12 months

The mean number of PCP consultations for LGB participants were significantly higher (GBM: 2.39 [95% CI 2.04–2.73]; LBW: 3.02 [2.64, 3.40]) compared to heterosexual men (1.91 [1.87–1.96]) but not heterosexual women (2.63 [2.58–2.69]) (Table 1 and Fig. 2).

### Full theoretical model and double-selection lasso findings

Compared with heterosexual men, the number of PCP consultations in the previous 12 months was higher among GBM (RR:1.29 [95% CI 1.12, 1.48] and LBW (RR:1.23 [1.10, 1.39]). Additionally, estimates between GBM, LBW, and heterosexual women were comparable (Tables 2 and 3). 

**Table 2:** Rate ratios (RR) for consultations with a primary care physician in the previous 12 months (2015–2016 CCHS; analytic sample = 82,294; weighted population = 23,295,391)

	**RR (95% CI)**
**Primary variables of interest**	
**Gay and bisexual**	
No	Reference
Yes	1.29 (1.12, 1.48)
**Sex**	
Male	Reference
Female	1.24 (1.20, 1.28)
**Interaction term**	
Heterosexual X male	Reference
Gay/bisexual X female	0.77 (0.65, 0.92)
**Covariates (predisposing, enabling, need-based and other health behaviours from BMHSU)**	
**Age (years)**	
18 – 29	Reference
30 – 39	1.02 (0.96, 1.08)
40 – 49	0.90 (0.85, 0.97)
50 – 59	0.89 (0.83, 0.95)
60 – 69	0.88 (0.82, 0.94)
≥70	0.94 (0.87, 1.01)
**Education**	
< Secondary education	Reference
Secondary and < post secondary education	0.98 (0.93, 1.04)
≥ Post-secondary education	1.02 (0.97, 1.07)
**Student**	
No	Reference
Yes	1.01 (0.94, 1.08)
**Racialized**	
No	Reference
Yes	1.08 (1.03, 1.14)
**Immigrant**	
No (Canadian born)	0.96 (0.92, 1.01)
Yes	Reference
**Marital status**	
Single	Reference
Married or common-law	1.14 (1.08, 1.21)
Divorced, widowed, or separated	1.09 (1.03, 1.15)
**Sense of belonging to local community**	
Very strong	Reference
Somewhat strong	0.94 (0.90, 0.98)
Somewhat to very weak	0.93 (0.88, 0.97)
**Geographical area**	
Atlantic	Reference
Quebec	0.57 (0.55, 0.60)
Ontario	0.84 (0.81, 0.88)
Prairies	0.94 (0.90, 0.98)
British Columbia	1.10 (1.05, 1.15)
Territories	0.95 (0.86, 1.04)
**Annual household income**	
< $40,000	Reference
$40,000 - $79,999	0.97 (0.93, 1.01)
≥$80,000	0.96 (0.91, 1.00)
**Regular health care provider**	
No	Reference
Yes	2.04 (1.92, 2.18)
**Insurance covers all or part of medication cost**	
No	Reference
Yes	1.14 (1.10, 1.19)
**Living arrangement**	
Living alone	Reference
Living with others	0.97 (0.93, 1.02)
**Self-perceived general health**	
Excellent or very good	Reference
Good	1.23 (1.18, 1.27)
Fair or poor	1.57 (1.48, 1.65)
**Self-perceived life stress**	
Not very or not at all stressful	Reference
A bit stressful	1.04 (1.01, 1.08)
Quite a bit or extremely stressful	1.12 (1.08, 1.17)
**Self-perceived mental health**	
Excellent or very good	Reference
Good	1.04 (1.00, 1.07)
Fair or poor	1.11 (1.07, 1.16)
**Chronic physical health conditions**	
None	Reference
One	1.33 (1.29, 1.38)
Two	1.53 (1.46, 1.60)
≥Three	1.69 (1.61, 1.78)
**Mood or anxiety disorder**	
No	Reference
Yes	1.42 (1.36, 1.48)
**Heavy drinker**	
No	Reference
Yes	0.97 (0.93, 1.01)
**E** **mergency room visits in the previous 12 months**	1.07 (1.06, 1.07)

Covariate estimates are included to maintain full reporting, but they are not substantively interpreted. Their role is limited to adjusting the associations for the primary variables

*BMHSU* Andersen’s Behavioural Model of Health Services Use

**Table 3:** Comparing rate ratios between estimates from the full theoretical model and those obtained using double-selection lasso regression

	Full theoretical model^a^ RR (95% CI)	Data-driven model	
		Double – selection lasso regression^b^ RR (95% CI)	Double - selection lasso regression with additional interaction terms^c^ RR (95% CI)
Heterosexual men	Reference	Reference	Reference
Heterosexual women	1.24 (1.20, 1.28)	1.24 (1.20, 1.28)	1.24 (1.20, 1.29)
Gay and bisexual men	1.29 (1.12, 1.48)	1.29 (1.12, 1.48)	1.29 (1.12, 1.49)
Lesbian and bisexual women	1.23 (1.10, 1.39)	1.23 (1.10, 1.39)	1.24 (1.10, 1.39)

95% CIs have been bootstrapped using 1000 sampling replicate weights provided by Statistics Canada; Rate ratios (RRs) are not statistically significantly different between heterosexual women and GBM, heterosexual women and LBW, and GBM and LBW

^a^When women on folic acid supplementation were excluded, the RR for heterosexual women decreased by 5%, for GBM by 1.5% and for LBW by 1.6% but remained significantly higher than heterosexual men (Supplementary Table 7b)

^b^Selection of the lambda parameter for lasso regression done using 10-fold cross-validation, adaptive lasso, and a plug-in formula. Results using different lambda selection methods are not presented separately as they yielded comparable results

^c^Additional interaction terms included:  Age X chronic physical health conditions, racial identity X annual household income, geographical regions X regular health care provider, mood or anxiety disorder X heavy drinker, immigrant X sense of belonging to local community, regular health care provider X insurance covers all or part of medication cost.

Double-selection lasso regression estimates were consistent with those of the full theoretical model, even when additional interaction terms were included (Table 3). This suggests that we could choose either to adjust for all covariates (as in the full theoretical model**)** or use a data-driven approach (double-selection lasso regression**)** to quantify the associations between the outcome and explanatory variables. Finally, our results also suggest that the interactions chosen to be included as covariates did not meaningfully affect the association (Table 3).

### Sensitivity analyses findings

Estimates of the expected number of PCP visits from the zero-inflated Poisson for GBM were similar to those of the full theoretical model; estimates for LBW were reduced by 12% though still significantly higher than heterosexual men. Overall, 97% of the participants with a regular health care provider reported that their provider was a PCP, and LBW were less likely to have a regular health care provider than heterosexual women (Table 1). Therefore, it is possible that some LBW reported zero consultations with a PCP because they did not have a regular PCP, which may also explain LBW’s higher emergency room visits (Table 1). Estimates from the full theoretical model were comparable to those when the definition of LGB was extended to include same-sex sexual behaviour. Extreme case imputations, wherein those participants excluded for missing sexual identity data were assumed to be either all LGB or all heterosexual, changed the estimates by less than 5%, suggesting that our primary findings are unaffected by their exclusion. Similarly, multiply imputing missingness in any of the 19 covariates changed estimates for the association of PCP consultations with LGB identity by < 1%. When HIV testing in the previous 12 months (with imputations) was also included, estimates for GBM were reduced by 9% and LBW by 4% indicating that some of the PCP consultations may be explained by visits for STBBI testing; nevertheless, the estimated number of PCP consultations remained significantly higher among GBM and LBW compared with heterosexual men.

Sensitivity analyses findings are presented in Supplementary Tables 2–5 and post-hoc analyses findings are discussed below.

## Discussion

Using a pan-Canadian dataset and both theoretically-informed and data-driven quantitative approaches, we found that GBM and LBW had higher rates of PCP consultations than heterosexual men over a one-year period. These differences persisted after accounting for multiple predisposing, enabling, and need-based factors. In addition, consultation rates for GBM and LBW were similar to those observed among heterosexual women.

Our finding that GBM consulted a PCP at higher rates than heterosexual men aligns with results from Tjepkema’s analysis of the 2003–2005 CCHS and a UK study using the English General Practice Survey [27, 37]. By comparison, our observation that LBW consulted at similar rates to heterosexual women differs from both studies, which reported lower consultation among LBW. However, comparability with our analysis is limited, as these earlier studies treated consultation as a binary (ever/never) outcome, stratified analyses by sex rather than modeling it as an interaction term, and adjusted for fewer factors.

Modelling the interaction between sexual identity and sex, rather than stratifying by sex as is conventional, allows us to use a single comparison group (heterosexual men) and interpret how these characteristics jointly relate to primary care utilization. In our analysis, this approach brings into focus gendered asymmetries that would remain obscured in within-sex comparisons. For example, a sex-stratified analysis would only show that LBW and heterosexual women have comparable consultation rates, obscuring that both groups of women in our sample consult more frequently than heterosexual men. Our findings allow us to postulate that, for LBW, the broader social position associated with being a woman may shape primary care utilization more than sexuality itself, whereas among GBM, sexuality may be a more salient factor. Thus this approach facilitates the generation of more nuanced hypotheses that move beyond examining biological sex as a binary driver of health care use to incorporate the social dimensions associated with sex, which can be explored in future work.

The higher consultation rates observed among GBM and LBW relative to heterosexual men may, in part, reflect the broader underutilization of primary care services by men. Numerous studies have documented that men generally engage less with primary care than women [38, 39]. This pattern has often been attributed to the notion that health-seeking behaviour conflicts with traditional masculine norms emphasizing autonomy and self-reliance [40]. Some researchers have suggested that GBM, on account of their sexuality, are less constrained by these gendered norms and therefore engage with health services more [41]. However, this assumes that sexual identity insulates GBM from the same gendered socialization processes that shape expectations for heterosexual men, an assumption that flattens the diversity and complexity of masculine identities. More recent research underscores this point, showing that masculinity is not a monolithic construct [42], and that, when examining comparable comorbidity profiles (men versus women), the apparent underutilization among men becomes less evident [39, 40].

In a post-hoc analysis stratifying by the number of chronic physical health conditions, we found that among individuals with at least one chronic condition, consultation rates for GBM and LBW were statistically comparable to those of heterosexual men (Supplementary Table 6b). Similarly, when the analysis was restricted to individuals with at least one consultation, estimates for heterosexual men and LBW were also comparable (Supplementary Table 6b). Although heterosexual men did consult less frequently than GBM and LBW among those without chronic conditions, the comparability observed when chronic conditions or existing engagement in care were taken into account suggests that the higher rates among GBM and LBW are not reducible to a universal underutilization of primary care services among heterosexual men.

Comparable PCP consultation rates observed among GBM, LBW, and heterosexual women represent a notable finding and further illustrate the utility of using a single reference group. The analysis makes visible not only that both heterosexual women and LBW consult more frequently than heterosexual men (noted above), but also that GBM consultation rates more closely resemble those of women than those of heterosexual men. This cross-cutting finding would be less transparent in sex-stratified models that rely on separate reference groups for men and women. While this relational finding is analytically apparent, it does not imply a shared underlying mechanism. Predisposing, enabling, and need-based factors posited in the BMHSU are distributed differently among heterosexual women, LBW, and GBM (Supplementary Table 8), suggesting that comparable consultation rates may arise through distinct pathways. Clarifying these pathways will require more granular data on gendered norms, sexuality-related health needs, and structural conditions shaping primary care engagement.

Our analyses could not adjust for *perceived quality of care*, a dimension of unmet health need not measured in the CCHS. As described by Allin et al., when perceived quality is unaccounted for in analytical models, the additional consultations observed in individuals dissatisfied with their care are misattributed to group differences rather than to deficiencies in care quality [43]. This omission may produce a counterintuitive analytical artefact: higher consultation rates may appear among groups that actually experience poorer care, reflecting residual variation in care quality rather than genuine differences in use. Therefore, our finding of higher PCP consultation rates among GBM and LBW relative to heterosexual men could, in part, reflect non-adjustment for substandard quality of care.

Canadian studies consistently document experiences of substandard care among sexual minority patients in primary care settings. Reviews of LGBTQ+ (lesbian, gay, bisexual, transgender, queer +) patient experiences in Canada have identified persistent heteronormativity and lack of inclusivity in clinical encounters, limited provider knowledge and competence regarding sexual minority health, and, at times, overt discrimination [23, 44]. In Nova Scotia, more than one-third of LGB respondents reported at least one negative experience within the health system [45]. Disclosure of sexual identity to primary care providers also remains uneven and is often shaped by patients’ concerns about discrimination or receiving poorer care following disclosure. In an Ontario study, only about half of GBM reported that their PCP was aware of their sexuality [46], while findings from the OutLook Study showed that even when disclosure occurs, many patients still feel uncomfortable discussing sexuality-related health care needs with most of their PCPs [47].

These studies highlight enduring provider-level barriers to equitable care, informed and compounded by limited formal training on sexual and gender minority health within medical education and continuing professional development [44]. Recent data from Canada indicate that undergraduate four-year medical programs include a median of only 12 h of LGBTQ+ health content [48]. However, these blind spots in provider knowledge and institutional preparedness extend beyond individual clinicians and medical institutions to the policy level. A review of 224 Canadian primary care policy documents found that only five acknowledged inequities faced by sexual and/or gender minorities, and none proposed concrete solutions [49].

Lastly, beyond potential analytical artefacts arising from the inability to account for care quality, our findings may also be shaped by fragmented care [50, 51]. Given documented experiences of substandard and often exclusionary care for sexual minorities, GBM and LBW may separate their sexual and non-sexual health consultations, seeking routine care from their regular PCP and sexual health care from another provider. In Canada, many sexual health clinics are staffed by PCPs with additional training in sexual health or who identify as part of the LGBTQ+ community. These clinics may be perceived as more inclusive, which could encourage GBM and LBW to seek sexual health care there more frequently. This pattern of fragmenting care could contribute to the higher PCP consultation rates observed among GBM and LBW. However, this interpretation should be viewed cautiously, as available data do not allow us to determine whether patients identify physicians at sexual health clinics as PCPs. Care fragmentation has been described more extensively among GBM in the United States [50, 51], and it would be useful for future studies to examine its relevance within Canada’s primary care system.

There are several limitations to our findings. First, we grouped bisexual and gay/lesbian individuals together to improve statistical power. However, bisexual individuals may have distinct patterns of health care engagement compared with gay and lesbian individuals. Thus, our estimates may obscure important differences within sexual minority groups. Additionally, we were unable to account for how sexual identity intersects with other social positions such as racial identity, immigration status, and socioeconomic disadvantage. The number of LGB respondents in the CCHS is already comparatively small, and further stratification by these characteristics is not feasible, precluding meaningful intersectional analyses. As a result, within-group heterogeneity among GBM and LBW is likely obscured. In addition, because the 2015–2016 CCHS did not collect data on gender identity, we were unable to distinguish participants by gender identity. As a result, our analyses do not allow for examination of PCP consultation rates among transgender or gender-diverse individuals. These limitations underscore the importance of the 2019 Parliamentary Standing Committee on Health’s recommendation to oversample sexual and gender minority populations in national surveys to ensure adequate representation [52]. Second, the cross-sectional design of the CCHS prevents us from evaluating continuity of care or temporal patterns of engagement, which may differ between GBM and LBW and may relate to downstream health outcomes. Longitudinal studies are needed to clarify these trajectories. Third, our outcome is self-reported and may be subject to recall, minimization, or maximization biases. Nonetheless, because sexual identity remains largely unrecorded in medical records across Canada [53], the CCHS remains one of the strongest datasets available for examining PCP consultations by sexual identity. Fourth, although our dataset predates the COVID-19 pandemic, from 2017 the CCHS restricted PCP consultation questions to mental health-related visits. The 2015–2016 cycle therefore provides the most recent nationally representative data on PCP consultations for all health reasons. Lastly, best statistical practices for applying bootstrap replicate weights in the context of double-selection lasso are not yet available [54], and findings from the lasso models should be interpreted with this limitation in mind.

## Conclusion

Even after adjusting for key predisposing, enabling, and need-based factors, GBM and LBW had higher PCP consultation rates than heterosexual men. These differences are unlikely to be explained solely by lower primary care engagement among heterosexual men. Instead, they may reflect limitations of the survey data, particularly the inability to account for perceived quality of care, and the possibility that substandard or exclusionary care contributes to fragmented care-seeking among sexual minorities. Consultation rates for GBM and LBW were comparable to those of heterosexual women, but without more detailed data, the mechanisms underlying this comparability remain unclear and warrant further investigation.

Canada’s primary care system is undergoing significant reform. However, without explicit inclusion of sexual identity in primary care planning and policy design, these reforms risk entrenching existing inequities. More comprehensive, timely, and disaggregated data, including patient-reported assessments of care quality, are needed to support the development of more inclusive models of primary care.

## Supplementary Information

Supplementary Material 1.

## Data Availability

Data used are publicly available to individuals affiliated with Canadian universities through Odesi, a data repository (https://odesi.ca/en). Others may contact Statistics Canada at [https://www150.statcan.gc.ca/n1/en/catalogue/82M0013X2019001].

## Abbreviations

BMHSU: Behavioural Model of Health Services Use
CCHS: Canadian Community Health Survey
GBM: Gay and Bisexual Men
HIV: Human Immunodeficiency Virus
LBW: Lesbian and Bisexual Women
LGB: Lesbian, Gay, and Bisexual
PCP: Primary Care Physician
PUMF: Public Use Microdata File
STBBI: Sexually Transmitted and Bloodborne Infections
