# Supplementary material for: Evaluating primary health care utilization among lesbian, gay, and bisexual individuals in Canada with a view to inform more equitable health measures
Source: BMC Public Health. 2026 Mar 9;26:1253. doi: 10.1186/s12889-026-26949-6 (PMC13085686; doi:10.1186/s12889-026-26949-6)
Supplement: Supplementary file 1 — Supplementary Material 1. [file 12889_2026_26949_MOESM1_ESM.docx]

**Supplementary Section**

**A: Lasso and double-selection lasso**

1. **Lasso regression**

Least absolute shrinkage and selection operation (lasso) is a type of penalized regression [1, 2]. It shrinks the estimated regression coefficients of a set of variables closer to the null (zero), thereby retaining stronger predictors and omitting weaker predictors (by shrinking their coefficients to zero) [2]. This property allows it to be used for variable selection.

For a Poisson model of the form:

$\log(E\left( Y=y | X=x \right))= \beta_{0}+\sum_{j=1}^{p} \beta_{j}x_{ij}$,

where $log(E\left( Y=y | X=x \right))$ is the natural logarithm of the modelled mean for outcome variable $Y$; $p$ the number of predictors, indexed by $j$; $\beta_{0}$ is the “baseline” mean outcome representing the expected value of $Y$ when all covariates $x$ take value 0; $N$ the number of observations; $\beta_{j}$ the coefficient associated with predictor $j$ representing the rate ratio associated with a 1-unit change in $x$; and $x_{ij}$is the $i^{th}$ value of variable $x$ for predictor $j$.

Lasso regression minimizes the function in the expression below, and thus generates estimates for $\hat{\beta}_{\lambda}^{lasso}$ [2-4]:

.

Negative log-likelihood

Sum of coefficients

**Penalization parameter (lambda) > 0**

$$-\frac{1}{N}\sum_{i=1}^{N} \left( y_{i}(\beta_{0}+\sum_{j=1}^{p} \beta_{j}x_{ij})-{exp}^{\beta_{0}+ \sum_{j=1}^{p} \beta_{j}x_{ij}} \right) + \boldsymbol{\lambda}\times\sum_{j=1}^{p} |\beta_{j}|$$

If λ is set to zero, the model reduces to the negative log-likelihood for a standard (unpenalized) Poisson model. Note that for every λ there is a $\beta$, some of the coefficients $\beta_{j}$ may be zero, and as λ increases the number of nonzero coefficients decreases (i.e., higher penalization leads to retaining fewer nonzero coefficients) [2]. However, an optimal λ is that for which the set of predictors with nonzero coefficients minimizes the expression above [1].

**Methods for estimating λ (limited to the current analyses)**

1. **10-fold cross-validation:** Cross-validation finds λ that produces coefficients that provide the best out-of-sample predictions [2]. The process for 10-fold cross validation is briefly described. First, the value of λ when all coefficients are penalized to zero is identified ($\lambda_{1}$) (i.e., the value of λ that omits all coefficients) [2]. Next, a grid of λ values is created, starting with $\lambda_{1}$and decreased stepwise by uniform logarithmic intervals (usually the grid has 100 λ values, but it may contain less or more) [2]. The dataset is then divided randomly into 10 folds [2]. One fold is chosen at a time, and a regression model (Poisson regression in our case) is fit on the other 9 folds using the variables in the model, for λ grid values [2]. Using the new coefficients obtained, predictions for the data in the fold chosen are computed [2]. For Poisson models, the deviance of the predictions is then computed [2]. This process is repeated for the remaining 9 folds [2]. Deviances over the 10 folds are averaged and the optimal λ is that which is associated with the lowest mean deviance (called cross-validation function)[2]. Supplementary Figure 1 portrays the relationship between the cross-validation function and λ values for predictors of the outcome (number of primary care physician (PCP) consults in the previous 12 months) in the present analysis.

**Supplementary Figure 1: Cross-validation plot**


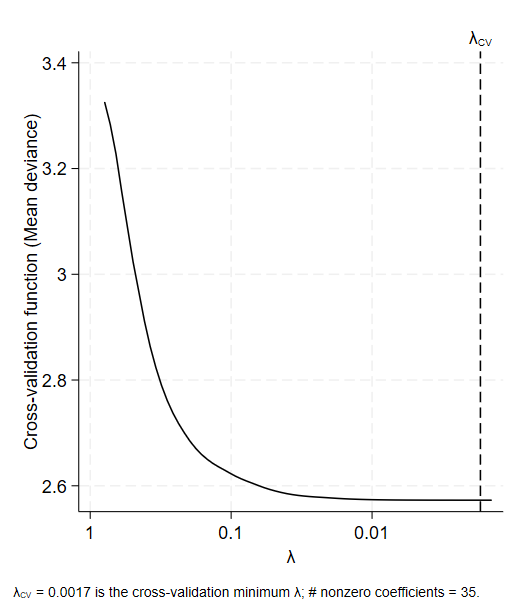


1. **Adaptive lasso:** Adaptive lasso is a weighted version of lasso [2, 5, 6]. While in traditional lasso regression all the coefficients are reduced by a similar magnitude, adaptive lasso uses weights to amplify important coefficients and attenuate unimportant ones [2]. Adaptive lasso first estimates coefficient values using standard unpenalized regression (commonly done, though a penalized ridge regression may also be used) [5]. The absolute coefficient values are then used as inverse weights to adjust for λs (which can be obtained using cross-validation). The weights tend to drive smaller coefficients closer to zero [2]. Unlike the traditional lasso, adaptive lasso has the oracle property, i.e., it consistently selects the correct predictors of the outcome and it demonstrates asymptotic normality in coefficient estimation [5, 6].

$$-\frac{1}{N}\sum_{i=1}^{N} \left( y_{i}(\beta_{0}+\sum_{j=1}^{p} \beta_{j}x_{ij})-{exp}^{\beta_{0}+ \sum_{j=1}^{p} \beta_{j}x_{ij}} \right) + \boldsymbol{\lambda}\times\sum_{j=1}^{p} \frac{1}{w_{j}}|\beta_{j}|$$

**weights**

1. **Plug-in formula for λ**: Using the equation or plug-in formula presented below directly and an iterative algorithm [2], the optimal value of **λ c**an be obtained. Please refer to Drukker et al.[7] for details on implementation.

$$\lambda= \frac{c}{\sqrt{N}} \Phi^{-1}(1-\frac{\gamma}{2p})$$

where, $c$ is a constant set at 1.1 [2, 8], $N$ is the sample size, $\Phi^{-1}$is the inverse of the cumulative density function for a normal distribution, $\gamma=0.1/ln[\max\left\{ p,N \right\}]$ is the probability of not removing a variable when it has a coefficient of zero, and $p$ is the number of predictors in the model [2, 7, 8].

Note all variables are standardized before they are used in lasso regression, so that coefficient sizes can be compared easily and are not dependent on the units of variables. The plug-in formula tends to select the fewest variables, compared to cross-validation and adaptive lasso [2]. Options for lasso with 10-fold cross validation, adaptive lasso, and plug-in formula can be directly specified in Stata 18.0 commands.

1. **Double-selection lasso regression:** When the objective of an analysis is to evaluate the association between a select number of variables (such as PCP consultations in the previous 12 months and sexual identity, as in our analysis), identifying appropriate adjustment variables (called *adjustment factors* in our analysis) for this association may not always be evident from existing literature [9-11]. The exclusion of adjustment variables that predict the outcome (dependent) variable and are correlated with the focal predictor(s) (explanatory variable in our analysis) may result in the estimated coefficients being either artificially strong or suppressed [9]. Double-selection lasso uses data-dependent selection, operationalized in two main steps to help identify appropriate adjustment variables [2, 9-11]:
2. Using lasso, a set of variables that predict the outcome variable is selected (i.e., variables with nonzero coefficients in lasso regression).
3. Using lasso again, a set of variables that predict the explanatory variable is selected.

The association between the outcome and explanatory variables is then estimated using a standard (unpenalized) regression model of the outcome variable on the explanatory variable, with the union of variables in (i) and (ii) serving as adjustment variables. Implementing the two steps reduces the probability of *omitted-variable bias* by ensuring that no variables which may be strong predictors of the explanatory variable but weak to moderate predictors of the outcome variable are omitted [10, 11]. Further, running the two steps ideally keeps the residual variance small [10].

Double-selection lasso provides unbiased estimates (unlike a single-step lasso where coefficients are biased towards the null) and allows statistical inferences (through the construction of confidence intervals) to be made for the association between the outcome and explanatory variable(s) [9-11]. Double-selection lasso works in low-dimensional settings (as is the case with our dataset where the number of observations is greater than the number of predictors) and in high dimensional approximately sparse settings (i.e., when the number of observations is close to or less than the number of predictors, but where a smaller subset of predictors can be identified reasonably) [12].

1. **Double-selection lasso regression as is related to the present analyses**
2. **Rationale for use of double-selection lasso regression:** Although Andersen’s Behavioural Model of Health Services Use (BMHSU) guided the identification of adjustment variables for the association between PCP consultations and sexual identity, we used double-selection lasso to investigate:
3. If a more parsimonious model with a fewer number of adjustment variables could be identified, which could facilitate easier/faster/less resource intensive data collection in future (through the collection of a smaller set of variables);
4. If interactions between adjustment variables significantly affected the association, which could be important research directions to consider.
5. **Implementation of the double-selection lasso regression**

$$\boldsymbol{log}\left( \boldsymbol{E}\left( \boldsymbol{Y} | \boldsymbol{X,A} \right) \right)\boldsymbol{=}\boldsymbol{\beta}_{\boldsymbol{0}}\boldsymbol{+}\boldsymbol{\beta}_{\boldsymbol{1}}\boldsymbol{gay\_bisexual +}\boldsymbol{\beta}_{\boldsymbol{2}}\boldsymbol{female +}\boldsymbol{\beta}_{\boldsymbol{3}}\boldsymbol{gay\_bisexual*female+}\boldsymbol{\beta}_{\boldsymbol{p}}\boldsymbol{covariates}$$

In the Poisson model of the form specified above, our objective was to evaluate the association between the mean of outcome ($Y)$: PCP consultations (count data, minimum 0, maximum 31) in the previous 12 months, and explanatory variable $(X)$: sexual identity (denoted by the binary variable *gay_bisexual*, where 0 = heterosexual and 1 = Gay and bisexual). We wanted to estimate the association separately for men and women, for which we include the binary variable *female* (0=male, 1=female) and the interaction term *gay_bisexual*female*. Adjustment factors $(A)$ are presented in Supplementary Table 6.1.

Thus, for the same adjustment factor values and relative to heterosexual men, ${exp}^{\beta_{1}}$represents the rate ratio for the average number of PCP consultations for gay and bisexual men, ${exp}^{\beta_{2}}$ the rate ratio for the average number of consultations for heterosexual women, and, ${exp}^{\beta_{1}+\beta_{2}+\beta_{3}}$ the rate ratio for the average number of consultations for lesbian and bisexual women.

Double-selection lasso was implemented using the following steps:

1. Adjustment factors from the set $A$ that predicted $Y$were selected.
2. Adjustment factors from the set $A$ that predicted $X$ were selected.
3. Adjustment factors from the set $A$ that predicted $female$ were selected.
4. Adjustment factors from the set $A$ that predicted $gay\_bisexual*female$ were selected.
5. A union of the variables selected in (1) to (4) was used as adjustment factors in a regular Poisson regression model to assess the association of interest.

Steps 3 and 4 were performed to allow the assessment of the association of interest separately by sex and sexual identity. Given that we used survey data, survey weights were incorporated in all regressions (including all lasso regressions 1 to 4).

Although double-selection lasso identified between 35 to 55 adjustment factors (**Supplementary Table 6.1**), the covariates themselves were largely unchanged which may explain the consistency in estimates between the full theoretical and data-dependent selection models (**Table 6.3, main manuscript**). (As noted, the adjustment factors include separate indicators for each level of a multi-category variable, and the set of double-selection lasso variables may include some but not all indicators for a given covariate).

**B: Supplementary Tables**

**Note on why all levels of categorical variables are presented**: Lasso treats categorical (variables with more than 2 levels) variables differently from traditional estimators [2]. With lasso, we specify variables that might be included in the model and a data-dependent process selects variables to be included. When specifying variables, lasso must be presented with all possible alternatives [2]. Thus, when including categorical variables, it is important to include the full set of indicators [2]. If the full set of indicators is not included (i.e., if we set the basal/reference level of the categorical variables and exclude it from the set of variables for selection), lasso is denied the possibility of identifying a more parsimonious model, by requiring it to select two or more variables for prediction that could have been accounted for by the excluded level [2].

| **Supplementary Table 1: The union of *adjustment factors* used in double-selection lasso regression** | | | | | |
| --- | --- | --- | --- | --- | --- |
|  | | **Adjustment factors selected ^a^** | | | |
|  |  | **Model with no interaction terms** | | | **Model with interaction terms** |
|  |  | **λ selected using** | | | |
|  |  | **10-fold cross-validation** | **Adaptive lasso** | **Plug-in formula** | **Plug-in formula ^b^** |
| 1 | Age category 18 – 29 years | X | X | X | X |
|  | Age category 30 – 39 years | X | X | X | X |
|  | Age category 40 – 49 years |  |  |  |  |
|  | Age category 50 – 59 years | X | X |  |  |
|  | Age category 60 – 69 years | X | X |  |  |
|  | Age category ≥70 years | X | X | X | X |
| 2 | Education <Secondary education | X | X | X |  |
|  | Education Secondary and <post-secondary education | X | X |  |  |
|  | Education ≥ Post-secondary education | X | X | X | X |
| 3 | Student | X | X | X | X |
| 4 | Racialized | X | X | X | X |
| 5 | Citizen | X | X | X | X |
| 6 | Marital status – single | X | X | X | X |
|  | Marital status – married/common-law | X | X | X | X |
|  | Marital status – Divorced, widowed, or separated | X | X | X | X |
| 7 | Geographical region – Atlantic | X | X | X |  |
|  | Geographical region – Quebec | X | X | X | X |
|  | Geographical region – Ontario | X | X | X | X |
|  | Geographical region – Prairies | X | X | X | X |
|  | Geographical region – British Columbia | X | X | X | X |
|  | Geographical region – Territories | X |  |  |  |
| 8 | Sense of belonging to local community – very strong | X | X | X | X |
|  | Sense of belonging to local community – somewhat strong | X | X |  |  |
|  | Sense of belonging to local community – somewhat to very weak | X | X | X | X |
| 9 | Heavy drinker | X | X | X | X |
| 10 | Household annual income < $40,000 | X | X | X | X |
|  | Household annual income $40,000 - $79,999 | X |  |  |  |
|  | Household annual income ≥$80,000 | X | X | X | X |
| 11 | Regular health care provider | X | X | X | X |
| 12 | Insurance covers all or part of medication cost | X | X | X |  |
| 13 | Living with others | X | X | X | X |
| 14 | Self-perceived general health – Excellent or very good | X | X | X | X |
|  | Self-perceived general health – Good | X | X |  |  |
|  | Self-perceived general health – Fair to poor | X | X | X | X |
| 15 | Self-perceived life stress – Not very or not at all stressful | X | X | X | X |
|  | Self-perceived life stress – A bit stressful |  |  |  |  |
|  | Self-perceived life stress – Quite a bit or extremely stressful | X | X | X | X |
| 16 | Self-perceived mental health – Excellent or very good | X | X | X | X |
|  | Self-perceived mental health – Good | X | X |  |  |
|  | Self-perceived mental health – Fair or poor | X | X | X | X |
| 17 | Chronic physical health conditions – None | X | X | X | X |
|  | Chronic physical health conditions – One |  |  |  |  |
|  | Chronic physical health conditions – Two | X | X | X | X |
|  | Chronic physical health conditions - ≥3 | X | X | X | X |
| 18 | Mood or anxiety disorder | X | X | X | X |
| 19 | Emergency room visits in the past 12 months | X | X | X | X |
| **Interaction terms** | | | | | |
| 20 | Age category 18 – 29 years with no chronic physical health conditions |  | | |  |
|  | Age category 18 – 29 years with 1 chronic physical health conditions |  |  |  |  |
|  | Age category 18 – 29 years with 2 chronic physical health conditions |  |  |  |  |
|  | Age category 18 – 29 years with ≥3 chronic physical health conditions |  |  |  |  |
|  | Age category 30 – 39 years with no chronic physical health conditions |  |  |  | X |
|  | Age category 30 – 39 years with 1 chronic physical health conditions |  |  |  |  |
|  | Age category 30 – 39 years with 2 chronic physical health conditions |  |  |  |  |
|  | Age category 30 – 39 years with ≥3 chronic physical health conditions |  |  |  |  |
|  | Age category 40 – 49 years with no chronic physical health conditions |  |  |  | X |
|  | Age category 40 – 49 years with 1 chronic physical health conditions |  |  |  | X |
|  | Age category 40 – 49 years with 2 chronic physical health conditions |  |  |  | X |
|  | Age category 40 – 49 years with ≥3 chronic physical health conditions |  |  |  | X |
|  | Age category 50 – 59 years with no chronic physical health conditions |  |  |  | X |
|  | Age category 50 – 59 years with 1 chronic physical health conditions |  |  |  |  |
|  | Age category 50 – 59 years with 2 chronic physical health conditions |  |  |  | X |
|  | Age category 50 – 59 years with ≥3 chronic physical health conditions |  |  |  |  |
|  | Age category 60 – 59 years with no chronic physical health conditions |  |  |  | X |
|  | Age category 60 – 69 years with 1 chronic physical health conditions |  |  |  | X |
|  | Age category 60 – 69 years with 2 chronic physical health conditions |  |  |  |  |
|  | Age category 60 – 69 years with ≥3 chronic physical health conditions |  |  |  |  |
|  | Age category ≥70 years with no chronic physical health conditions |  |  |  | X |
|  | Age category ≥70 years with 1 chronic physical health conditions |  |  |  |  |
|  | Age category ≥70 years with 2 chronic physical health conditions |  |  |  |  |
|  | Age category ≥70 years with ≥3 chronic physical health conditions |  |  |  | X |
| 21 | White with household annual income < $40,000 |  |  |  | X |
|  | White with household annual income $40,000 - $79,999 |  |  |  |  |
|  | White with household annual income ≥$80,000 |  |  |  | X |
|  | Racialized with household annual income < $40,000 |  |  |  | X |
|  | Racialized with household annual income $40,000 - $79,999 |  |  |  | X |
|  | Racialized with household annual income ≥$80,000 |  |  |  |  |
| 22 | Geographical region – Atlantic with no regular health care provider |  |  |  |  |
|  | Geographical region – Atlantic with a regular health care provider |  |  |  | X |
|  | Geographical region – Quebec with no regular health care provider |  |  |  |  |
|  | Geographical region – Quebec with a regular health care provider |  |  |  |  |
|  | Geographical region – Ontario with no regular health care provider |  |  |  |  |
|  | Geographical region – Ontario with a regular health care provider |  |  |  |  |
|  | Geographical region – Prairies with no regular health care provider |  |  |  | X |
|  | Geographical region – Prairies with a regular health care provider |  |  |  | X |
|  | Geographical region – British Columbia with no regular health care provider |  |  |  |  |
|  | Geographical region – British Columbia with a regular health care provider |  |  |  |  |
|  | Geographical region – Territories with no regular health care provider |  |  |  |  |
|  | Geographical region – Territories with a regular health care provider |  |  |  |  |
| 23 | Mood or anxiety disorder with heavy drinking |  |  |  | X |
| 24 | Regular health care provider with insurance |  |  |  | X |
| 25 | Immigrant with very strong sense of belonging to local community |  |  |  | X |
|  | Citizen with very strong sense of belonging to local community |  |  |  |  |
|  | Immigrant with somewhat strong sense of belonging to local community |  |  |  | X |
|  | Citizen with somewhat strong sense of belonging to local community |  |  |  | X |
|  | Immigrant with somewhat to very weak sense of belonging to local community |  |  |  |  |
|  | Citizen with somewhat to very weak sense of belonging to local community |  |  |  |  |
|  | **Total number of adjustment factors selected/ Total number of adjustment factors** | 43/46 | 41/46 | 35/46 | 55/96 |
|  | **Total number of covariates selected/Total covariates** | 19/19 | 19/19 | 19/19 | 24/25 |
| ^a^ The union of adjustment factors using double-selection lasso.  ^b^ Only plug-in formula shown for the model with interaction terms. Plug-in yielded the lowest number of predictors while producing estimates consistent with those for cross-validation and adaptive lasso. | | | | | |

References/reasoning for interaction terms included in the double-selection lasso regression

| **Interaction term** | **Reference/reasoning** |
| --- | --- |
| Age and number of chronic physical health conditions | Han BH, Duncan DT, Arcila-Mesa M, Palamar JJ. Co-occurring mental illness, drug use, and medical multimorbidity among lesbian, gay, and bisexual middle-aged and older adults in the United States: a nationally representative study. *BMC Public Health* 2020; **20**(1): 1123. |
| Racial identity and income | Block S, Galabuzi G-E, Tranjan R. Canada's Colour Coded Income Inequality. 2019. https://policyalternatives.ca/sites/default/files/uploads/publications/National%20Office/2019/12/Canada%27s%20Colour%20Coded%20Income%20Inequality.pdf |
| Geographic region and having a regular health care provider | Statistics Canada. Primary health care providers, 2019. 2020. https://www150.statcan.gc.ca/n1/en/pub/82-625-x/2020001/article/00004-eng.pdf?st=WoxZ-OPp |
| Having a mood or anxiety disorder and heavy drinking | Pakula B, Shoveller J, Ratner PA, Carpiano R. Prevalence and Co-Occurrence of Heavy Drinking and Anxiety and Mood Disorders Among Gay, Lesbian, Bisexual, and Heterosexual Canadians. Am J Public Health 2016; 106(6): 1042-8. |
| Canadian nativity and sense of belonging to the local community | Painter CV. Sense of belonging: Literature review. Citizenship and Immigration Canada. 2013. https://www.canada.ca/content/dam/ircc/migration/ircc/english/pdf/research-stats/r48a-2012belonging-eng.pdf |
| Regular health care provider and insurance coverage for medication | Included as an indicator for the perceived ease/difficulty of seeking PCP consultations – akin to the concept of *perceived behavioural control* in the Theory of Planned Behaviour. That is, individuals with both a regular provider and insurance coverage for medication are less likely to perceive accessibility or cost as barriers when deciding to consult a PCP, as compared to those who have neither, with varying degrees of perception in between). |

| **Supplementary Table 2a**: **Weighted distribution of counts for the outcome (i.e., consultations with a primary care physician in the previous 12 months) using data from the 2015- 2016 Canadian Community Health Survey** | | | | | |
| --- | --- | --- | --- | --- | --- |
| Number of consults | **Total**  % (95% CI) | **Men** | | **Women** | |
|  |  | Heterosexual  (95% CI) | Gay and bisexual  (95% CI) | Heterosexual  (95% CI) | Lesbian and bisexual  (95% CI) |
| 0 | 30.9 (30.5, 31.5) | 37.3 (36.6, 38.1) | 36.0 (30.9, 41.4) | 24. 8 (24.1, 25.5) | 25.2 (20.8, 30.1) |
| 1 | 21.7 (21.2, 22.1) | 21.6 (20.9, 22.2) | 17.2 (14.0, 20.9) | 22.0 (21.4, 22.6) | 19.7 (16.5, 23.3) |
| 2 | 16.6 (16.2, 17.0) | 15.5 (14.9, 16.2) | 18.7 (15.0, 23.0) | 17.5 (17.0, 18.1) | 17.3 (13.7, 21.6) |
| 3 | 10.0 (9.7, 10.3) | 9.1 (8.6, 9.6) | 6.2 (4.6, 8.3) | 10.9 (10.5, 11.4) | 11.6 (9.2, 14.5) |
| 4 | 8.1 (7.8, 8.4) | 7.1 (6.7, 7.5) | 7.2 (5.3, 9.8) | 9.1 (8.7, 9.6) | 7.4 (5.8, 9.3) |
| 5 | 3.1 (2.9, 3.3) | 2.4 (2.1, 2.6) | 3.4 (2.1, 5.4) | 3.8 (3.6, 4.1) | 4.4 (2.8, 6.7) |
| 6 | 3.3 (3.1, 3.4) | 2.4 (2.2, 2.6) | 3.2 (1.7, 5.9) | 4.0 (3.8, 4.3) | 4.6 (3.2, 6.4) |
| ≥7 | 6.3 (6.0, 6.6) | 4.6 (4.3, 4.9) | 8.1 (5.8, 11.2) | 7.8 (7.4, 8.2) | 9.9 (7.6, 12.8) |
| Range of visits reported: 0-31. Proportions for number of visits ≥7 not shown separately as most are <1%. | | | | | |

| **Supplementary Table 2b: Rate ratios for the number of consultations with a primary care physician in the previous 12 months** | |
| --- | --- |
|  | **Zero-inflated Poisson**  **RR (95% CI)** |
| **Gay and bisexual**  No  Yes | Reference  1.28 (1.12, 1.46) |
| **Sex**  Male  Female | Reference  1.13 (1.09, 1.16) |
| **Interaction term**  Heterosexual X male  Gay/bisexual X female | Reference  0.75 (0.63, 0.89) |
| **Age (years)**  18 – 29  30 – 39  40 – 49  50 – 59  60 – 69  ≥70 | Reference  0.98 (0.92, 1.05)  0.86 (0.80, 0.92)  0.80 (0.75, 0.86)  0.78 (0.72, 0.84)  0.82 (0.76, 0.88) |
| **Education**  < Secondary education  Secondary and < post secondary education  ≥ Post-secondary education | Reference  0.98 (0.92, 1.04)  0.97 (0.92, 1.02) |
| **Student**  No  Yes | Reference  0.96 (0.90, 1.03) |
| **Racialized**  No  Yes | Reference  1.09 (1.04, 1.15) |
| **Immigrant**  No (Canadian born)  Yes | 0.97 (0.93, 1.02)  Reference |
| **Marital status**  Single  Married or common-law  Divorced, widowed, or separated | Reference  1.08 (1.02, 1.15)  1.06 (1.00, 1.12) |
| **Geographical area**  Atlantic  Quebec  Ontario  Prairies  British Columbia  Territories | Reference  0.58 (0.55, 0.61)  0.88 (0.85, 0.93)  0.95 (0.90, 0.99)  1.07 (1.02, 1.13)  0.93 (0.85, 1.01) |
| **Sense of belonging to local community**  Very strong  Somewhat strong  Somewhat to very weak | Reference  0.92 (0.89, 0.96)  0.94 (0.89, 0.98) |
| **Household annual income**  < $40,000  $40,000 - $79,999  ≥$80,000 | Reference  0.95 (0.91, 0.99)  0.91 (0.87, 0.96) |
| **Regular health care provider**  No  Yes | Reference  1.22 (1.14, 1.31) |
| **Insurance covers all or part of medication cost**  No  Yes | Reference  1.09 (1.04, 1.13) |
| **Living arrangement**  Living alone  Living with others | Reference  1.00 (0.95, 1.05) |
| **Self-perceived general health**  Excellent or very good  Good  Fair or poor | Reference  1.24 (1.20, 1.29)  1.59 (1.50, 1.67) |
| **Self-perceived life stress**  Not very or not at all stressful  A bit stressful  Quite a bit or extremely stressful | Reference  1.02 (0.97, 1.05)  1.08 (1.04, 1.13) |
| **Self-perceived mental health**  Excellent or very good  Good  Fair or poor | Reference  1.01(0.97, 1.05)  1.16 (1.09, 1.24) |
| **Chronic physical health conditions**  None  One  Two  ≥Three | Reference  1.21 (1.16, 1.26)  1.36 (1.29, 1.43)  1.51 (1.43, 1.58) |
| **Mood or anxiety disorder**  No  Yes | Reference  1.30 (1.25, 1.35) |
| **Heavy drinker**  No  Yes | Reference  0.96 (0.92, 1.00) |
| **Emergency visits in the previous 12 months** | 1.06 (1.05, 1.07) |

| **Supplementary Table 2c: Odds ratios (ORs) for the excess number of zeros (logistic part of the Zero-inflated Poisson model)** | |
| --- | --- |
|  | **OR (95% CI)** |
| **Gay and bisexual**  No  Yes | Reference  1.02 (0.74, 1.40) |
| **Sex**  Male  Female | Reference  0.60 (0.55, 0.65) |
| **Interaction term**  Heterosexual X male  Gay/bisexual X female | Reference  0.83 (0.49, 1.41) |
| **Age (years)**  18 – 29  30 – 39  40 – 49  50 – 59  60 – 69  ≥70 | Reference  0.94 (0.82, 1.07)  0.85 (0.73, 0.98)  0.62 (0.52, 0.72)  0.51 (0.42, 0.61)  0.46 (0.38, 0.56) |
| **Education**  < Secondary education  Secondary and < post secondary education  ≥ Post-secondary education | Reference  0.91 (0.80, 1.04)  0.71 (0.62, 0.80) |
| **Student**  No  Yes | Reference  0.84 (0.71, 0.99) |
| **Racialized**  No  Yes | Reference  1.03 (0.89, 1.20) |
| **Immigrant**  No (Canadian born)  Yes | 1.05 (0.92, 1.20)  Reference |
| **Marital status**  Single  Married or common-law  Divorced, widowed, or separated | Reference  0.79 (0.69, 0.91)  0.91 (0.79, 1.05) |
| **Geographical area**  Atlantic  Quebec  Ontario  Prairies  British Columbia  Territories | Reference  1.00 (0.87, 1.15)  1.30 (1.15, 1.47)  1.02 (0.90, 1.15)  0.87 (0.75, 1.00)  0.92 (0.74, 1.15) |
| **Sense of belonging to local community**  Very strong  Somewhat strong  Somewhat to very weak | Reference  0.89 (0.80, 1.00)  1.01 (0.88, 1.14) |
| **Household annual income**  < $40,000  $40,000 - $79,999  ≥$80,000 | Reference  0.85 (0.76, 0.96)  0.72 (0.63, 0.81) |
| **Regular health care provider**  No  Yes | Reference  0.22 (0.20, 0.25) |
| **Insurance covers all or part of medication cost**  No  Yes | Reference  0.77 (0.70, 0.86) |
| **Living arrangement**  Living alone  Living with others | Reference  1.15 (1.02, 1.31) |
| **Self-perceived general health**  Excellent or very good  Good  Fair or poor | Reference  1.11 (1.01, 1.23)  1.17 (1.00, 1.37) |
| **Self-perceived life stress**  Not very or not at all stressful  A bit stressful  Quite a bit or extremely stressful | Reference  0.90 (0.82, 1.00)  0.87 (0.77, 0.98) |
| **Self-perceived mental health**  Excellent or very good  Good  Fair or poor | Reference  0.89 (0.80, 0.99)  0.79 (0.66, 0.96) |
| **Chronic physical health conditions**  None  One  Two  ≥Three | Reference  0.61 (0.55, 0.68)  0.50 (0.43, 0.58)  0.47 (0.40, 0.55) |
| **Mood or anxiety disorder**  No  Yes | Reference  0.52 (0.45, 0.59) |
| **Heavy drinker**  No  Yes | Reference  0.93 (0.84, 1.03) |
| **Emergency room visits in the previous 12 months** | 0.90 (0.86, 0.93) |

| **Supplementary Table 3a: Including same-sex sexual behaviours in the definition of LGB** | | | |
| --- | --- | --- | --- |
|  | **n** | **Weighted n** | **Weighted proportion (95% CI)** |
| Gay, bisexual, and other men who have sex with men (GBMSM) | 1,236 | 412,299 | 3.2 (2.9, 3.6) |
| Lesbian, bisexual, and other women who have sex with women (LBWSW) | 1,498 | 443,287 | 3.3 (3.1, 3.6) |

| **Supplementary Table 3b: Rate ratios for the number of consultations with a primary care physician in the previous 12 months including same sex behaviours** | |
| --- | --- |
|  | **RR (95% CI)** |
| **GBMSM**  No  Yes | Reference  1.29 (1.13, 1.47) |
| **Sex**  Male  Female | Reference  1.24 (1.20, 1.28) |
| **Interaction term**  Heterosexual X male  GBM X female | Reference  0.78 (0.66, 0.93) |
| **Age (years)**  18 – 29  30 – 39  40 – 49  50 – 59  60 – 69  ≥70 | Reference  1.02 (0.96, 1.09)  0.91 (0.85, 0.97)  0.89 (0.83, 0.95)  0.88 (0.82, 0.94)  0.94 (0.87, 1.01) |
| **Education**  < Secondary education  Secondary and < post secondary education  ≥ Post-secondary education | Reference  0.99 (0.94, 1.04)  1.02 (0.97, 1.07) |
| **Student**  No  Yes | Reference  1.00 (0.94, 1.07) |
| **Racialized**  No  Yes | Reference  1.08 (1.02, 1.14) |
| **Immigrant**  No (Canadian born)  Yes | 0.97 (0.93, 1.02)  Reference |
| **Marital status**  Single  Married or common-law  Divorced, widowed, or separated | Reference  1.15 (1.09, 1.22)  1.10 (1.04, 1.16) |
| **Geographical area**  Atlantic  Quebec  Ontario  Prairies  British Columbia  Territories | Reference  0.57 (0.55, 0.61)  0.84 (0.81, 0.88)  0.94 (0.90, 0.98)  1.10 (1.05, 1.15)  0.95 (0.86, 1.04) |
| **Sense of belonging to local community**  Very strong  Somewhat strong  Somewhat to very weak | Reference  0.94 (0.90, 0.98)  0.93 (0.88, 0.97) |
| **Household annual income**  < $40,000  $40,000 - $79,999  ≥$80,000 | Reference  0.97 (0.93, 1.01)  0.96 (0.91, 1.00) |
| **Regular health care provider**  No  Yes | Reference  2.04 (1.92, 2.18) |
| **Insurance covers all or part of medication cost**  No  Yes | Reference  1.14 (1.10, 1.19) |
| **Living arrangement**  Living alone  Living with others | Reference  0.97 (0.93, 1.02) |
| **Self-perceived general health**  Excellent or very good  Good  Fair or poor | Reference  1.23 (1.18, 1.27)  1.57 (1.48, 1.65) |
| **Self-perceived life stress**  Not very or not at all stressful  A bit stressful  Quite a bit or extremely stressful | Reference  1.04 (1.00, 1.07)  1.11 (1.07, 1.16) |
| **Self-perceived mental health**  Excellent or very good  Good  Fair or poor | Reference   - 1. (1.00, 1.07)   1.21 (1.14, 1.29) |
| **Chronic physical health conditions**  None  One  Two  ≥Three | Reference  1.33 (1.29, 1.38)  1.53 (1.46, 1.60)  1.69 (1.61, 1.78) |
| **Mood or anxiety disorder**  No  Yes | Reference  1.42 (1.36, 1.48) |
| **Heavy drinker**  No  Yes | Reference  0.97 (0.93, 1.02) |
| **Emergency room visits in the previous 12 months** | 1.07 (1.06, 1.07) |

| **Supplementary Table 4: Rate ratios for the number of consultations with a primary care physician in the previous 12 months** | | |
| --- | --- | --- |
|  | **Assuming all those with missing data for sexual identity are gay/lesbian and bisexual** | **Assuming all those with missing data for sexual identity are heterosexual** |
|  | **RR (95% CI)** | **RR (95% CI)** |
| **Gay and bisexual**  No  Yes | Reference  1.23 (1.10, 1.37) | Reference  1.29 (1.12, 1.48) |
| **Sex**  Male  Female | Reference  1.24 (1.20, 1.28) | Reference  1.24 (1.20, 1.27) |
| **Interaction term**  Heterosexual X male  Gay/bisexual X female | Reference  0.80 (0.69, 0.92) | Reference  0.77 (0.65, 0.92) |
| **Age (years)**  18 – 29  30 – 39  40 – 49  50 – 59  60 – 69  ≥70 | Reference  1.01 (0.95, 1.08)  0.90 (0.84, 0.96)  0.89 (0.83, 0.96)  0.87 (0.82, 0.93)  0.93 (0.86, 1.00) | Reference   - 1. (0.95, 1.08)   0.90 (0.84, 0.96)  0.89 (0.83, 0.95)  0.87 (0.82, 0.93)  0.93 (0.86, 1.00) |
| **Education**  < Secondary education  Secondary and < post secondary education  ≥ Post-secondary education | Reference  0.99 (0.94, 1.04)  1.02 (0.97, 1.07) | Reference  0.99 (0.94, 1.04)  1.01 (0.96, 1.06) |
| **Student**  No  Yes | Reference  1.01 (0.94, 1.08) | Reference  1.01 (0.94, 1.08) |
| **Racialized**  No  Yes | Reference  1.08 (1.03, 1.14) | Reference  1.08 (1.03, 1.14) |
| **Immigrant**  No (Canadian born)  Yes | 0.96 (0.92, 1.01)  Reference | 0.96 (0.92, 1.01)  Reference |
| **Marital status**  Single  Married or common-law  Divorced, widowed, or separated | Reference  1.15 (1.08, 1.21)  1.09 (1.03, 1.15) | Reference  1.15 (1.08, 1.21)  1.09 (1.03, 1.15) |
| **Geographical area**  Atlantic  Quebec  Ontario  Prairies  British Columbia  Territories | Reference  0.58 (0.55, 0.61)  0.84 (0.80, 0.88)  0.94 (0.89, 0.98)  1.10 (1.05, 1.16)  0.94 (0.86, 1.03) | Reference  0.58 (0.55, 0.61)  0.84 (0.80, 0.88)  0.94 (0.89, 0.98)  1.10 (1.05, 1.16)  0.94 (0.86, 1.03) |
| **Sense of belonging to local community**  Very strong  Somewhat strong  Somewhat to very weak | Reference  0.95 (0.91, 0.98)  0.93 (0.89, 0.98) | Reference  0.95 (0.91, 0.98)  0.93 (0.89, 0.98) |
| **Heavy drinker**  No  Yes | Reference  0.97 (0.93, 1.00) | Reference  0.96 (0.93, 1.00) |
| **Household annual income**  < $40,000  $40,000 - $79,999  ≥$80,000 | Reference  0.97 (0.93, 1.01)  0.97 (0.92, 1.01) | Reference  0.97 (0.93, 1.01)  0.96 (0.92, 1.01) |
| **Regular health care provider**  No  Yes | Reference  2.04 (1.92, 2.18) | Reference  2.04 (1.92, 2.18) |
| **Insurance covers all or part of medication cost**  No  Yes | Reference  1.14 (1.10, 1.19) | Reference  1.14 (1.10, 1.18) |
| **Living arrangement**  Living alone  Living with others | Reference  0.97 (0.92, 1.01) | Reference  0.97 (0.92, 1.01) |
| **Self-perceived general health**  Excellent or very good  Good  Fair or poor | Reference  1.23 (1.18, 1.27)  1.56 (1.48, 1.65) | Reference  1.23 (1.18, 1.27)  1.56 (1.48, 1.65) |
| **Self-perceived life stress**  Not very or not at all stressful  A bit stressful  Quite a bit or extremely stressful | Reference  1.04 (1.00, 1.07)  1.11 (1.07, 1.16) | Reference  1.04 (1.00, 1.07)  1.11 (1.07, 1.16) |
| **Self-perceived mental health**  Excellent or very good  Good  Fair or poor | Reference   - 1. (1.00, 1.07)   1.20 (1.13, 1.28) | Reference   - 1. (1.00, 1.07)   1.20 (1.13, 1.28) |
| **Chronic physical health conditions**  None  One  Two  ≥Three | Reference  1.34 (1.30, 1.39)  1.54 (1.47, 1.61)  1.70 (1.62, 1.78) | Reference  1.34 (1.29, 1.39)  1.54 (1.47, 1.61)  1.70 (1.62, 1.78) |
| **Mood or anxiety disorder**  No  Yes | Reference  1.42 (1.36, 1.48) | Reference  1.42 (1.36, 1.48) |
| **Emergency room visits in the previous 12 months** | 1.07 (1.06, 1.07) | 1.07 (1.06, 1.07) |

| **Supplementary Table 5a: Missingness distribution for imputed covariates, for Supplementary Tables 5b and 5c** | | | |
| --- | --- | --- | --- |
| **Variable** | **Complete** | **Imputed** | **Total** |
| **Student** | 93138 | 584 | 93722 |
| **Racialized** | 87967 | 5755 | 93722 |
| **Immigrant** | 92713 | 1009 | 93722 |
| **Mood or anxiety disorder** | 93558 | 164 | 93722 |
| **Heavy drinker** | 93135 | 587 | 93722 |
| **Insurance** | 93030 | 692 | 93722 |
| **Living arrangement** | 93245 | 477 | 93722 |
| **Regular health care provider** | 93643 | 79 | 93722 |
| **Education** | 92613 | 1109 | 93722 |
| **Household annual income** | 93605 | 117 | 93722 |
| **Marital status** | 93450 | 272 | 93722 |
| **Sense of belonging to local community** | 92547 | 1175 | 93722 |
| **Self-perceived general health** | 93596 | 126 | 93722 |
| **Self-perceived life stress** | 93353 | 369 | 93722 |
| **Self-perceived mental health** | 93578 | 144 | 93722 |
| **Chronic physical health conditions** | 93716 | 6 | 93722 |
| **Emergency room visits** | 93585 | 137 | 93722 |
| **HIV test in the previous 12 months** | 61370 | 32352 | 93722 |
| Complete + imputed = Total, 25 imputation sets using chained equations to obtain imputed values | | | |

| **Supplementary Table 5b: Rate ratios for the number of consultations with a primary care physician in the previous 12 months when missing values of covariates are imputed using chained equations (using 25 imputation sets)** | |
| --- | --- |
|  | **RR (95% CI)** |
| **Gay and bisexual**  No  Yes | Reference  1.30 (1.14, 1.48) |
| **Sex**  Male  Female | Reference  1.24 (1.20, 1.28) |
| **Interaction term**  Heterosexual X male  Gay/bisexual X female | Reference  0.76 (0.64, 0.90) |
| **Age (years)**  18 – 29  30 – 39  40 – 49  50 – 59  60 – 69  ≥70 | Reference  1.03 (0.97, 1.09)  0.91 (0.85, 0.98)  0.89 (0.84, 0.95)  0.88 (0.82, 0.94)  0.94 (0.87, 1.00) |
| **Education**  < Secondary education  Secondary and < post secondary education  ≥ Post-secondary education | Reference  0.98 (0.93, 1.03)  1.03 (0.98, 1.07) |
| **Student**  No  Yes | Reference  1.01 (0.95, 1.08) |
| **Racialized**  No  Yes | Reference  1.07 (1.02, 1.13) |
| **Immigrant**  No (Canadian born)  Yes | 0.96 (0.93, 1.00)  Reference |
| **Marital status**  Single  Married or common-law  Divorced, widowed, or separated | Reference  1.14 (1.08, 1.20)  1.08 (1.03, 1.14) |
| **Geographical area**  Atlantic  Quebec  Ontario  Prairies  British Columbia  Territories | Reference  0.58 (0.55, 0.61)  0.85 (0.82, 0.89)  0.96 (0.92, 1.00)  1.12 (1.07, 1.18)  0.86 (0.78, 0.95) |
| **Sense of belonging to local community**  Very strong  Somewhat strong  Somewhat to very weak | Reference  0.95 (0.91, 0.98)  0.94 (0.90, 0.98) |
| **Heavy drinker**  No  Yes | Reference  0.98 (0.95, 1.02) |
| **Household annual income**  < $40,000  $40,000 - $79,999  ≥$80,000 | Reference  0.97 (0.93, 1.01)  0.95 (0.91, 0.99) |
| **Regular health care provider**  No  Yes | Reference  2.09 (1.96, 2.23) |
| **Insurance covers all or part of medication cost**  No  Yes | Reference  1.14 (1.10, 1.19) |
| **Living arrangement**  Living alone  Living with others | Reference  0.97 (0.93, 1.02) |
| **Self-perceived general health**  Excellent or very good  Good  Fair or poor | Reference  1.21 (1.17, 1.25)  1.56 (1.48, 1.64) |
| **Self-perceived life stress**  Not very or not at all stressful  A bit stressful  Quite a bit or extremely stressful | Reference  1.04 (1.01, 1.07)  1.10 (1.06, 1.14) |
| **Self-perceived mental health**  Excellent or very good  Good  Fair or poor | Reference   - 1. (1.00, 1.07)   1.20 (1.13, 1.27) |
| **Chronic physical health conditions**  None  One  Two  ≥Three | Reference  1.34 (1.29, 1.39)  1.54 (1.47, 1.61)  1.72 (1.64, 1.81) |
| **Mood or anxiety disorder**  No  Yes | Reference  1.41 (1.36, 1.47) |
| **Emergency room visits in the previous 12 months** | 1.06 (1.05, 1.07) |

| **Supplementary Table 5c: Rate ratios for the number of consultations with a primary care physician in the previous 12 months when missing values of covariates including for HIV testing in the previous 12 months are imputed using chained equations (using 25 imputation sets)** | |
| --- | --- |
|  | **RR (95% CI)** |
| **Gay and bisexual**  No  Yes | Reference  1.17 (1.02, 1.33) |
| **Sex**  Male  Female | Reference  1.24 (1.20, 1.28) |
| **Interaction term**  Heterosexual X male  Gay/bisexual X female | Reference  0.82 (0.69, 0.96) |
| **Age (years)**  18 – 29  30 – 39  40 – 49  50 – 59  60 – 69  ≥70 | Reference  1.03 (0.97, 1.10)  0.94 (0.87, 1.00)  0.92 (0.86, 0.99)  0.92 (0.86, 0.98)  0.93 (0.86, 1.00) |
| **Education**  < Secondary education  Secondary and < post secondary education  ≥ Post-secondary education | Reference  0.99 (0.94, 1.04)  1.03 (0.98, 1.08) |
| **Student**  No  Yes | Reference  1.01 (0.94, 1.07) |
| **Racialized**  No  Yes | Reference  1.06 (1.01, 1.12) |
| **Immigrant**  No (Canadian born)  Yes | 0.97 (0.93, 1.01)  Reference |
| **Marital status**  Single  Married or common-law  Divorced, widowed, or separated | Reference  1.16 (1.09, 1.22)  1.08 (1.03, 1.14) |
| **Geographical area**  Atlantic  Quebec  Ontario  Prairies  British Columbia  Territories | Reference  0.57 (0.55, 0.60)  0.85 (0.81, 0.89)  0.95 (0.91, 0.99)  1.10 (1.05, 1.16)  0.85 (0.77, 0.93) |
| **Sense of belonging to local community**  Very strong  Somewhat strong  Somewhat to very weak | Reference  0.95 (0.92, 0.99)  0.94 (0.90, 0.98) |
| **Heavy drinker**  No  Yes | Reference  0.98 (0.94, 1.01) |
| **Household annual income**  < $40,000  $40,000 - $79,999  ≥$80,000 | Reference  0.97 (0.94, 1.02)  0.96 (0.92, 1.00) |
| **Regular health care provider**  No  Yes | Reference  2.08 (1.95, 2.21) |
| **Insurance covers all or part of medication cost**  No  Yes | Reference  1.14 (1.10, 1.18) |
| **Living arrangement**  Living alone  Living with others | Reference  0.98 (0.94, 1.02) |
| **Self-perceived general health**  Excellent or very good  Good  Fair or poor | Reference  1.21 (1.17, 1.25)  1.56 (1.48, 1.64) |
| **Self-perceived life stress**  Not very or not at all stressful  A bit stressful  Quite a bit or extremely stressful | Reference  1.04 (1.00, 1.07)  1.10 (1.06, 1.14) |
| **Self-perceived mental health**  Excellent or very good  Good  Fair or poor | Reference   - 1. (1.00, 1.07)   1.19 (1.13, 1.26) |
| **Chronic physical health conditions**  None  One  Two  ≥Three | Reference  1.34 (1.29, 1.39)  1.54 (1.47, 1.61)  1.72 (1.64, 1.80) |
| **Mood or anxiety disorder**  No  Yes | Reference  1.41 (1.35, 1.46) |
| **Emergency room visits in the previous 12 months** | 1.06 (1.05, 1.07) |
| **HIV testing in the previous 12 months ^a^**  No  Yes | Reference  1.35 (1.28, 1.41) |
| ^a^ No includes those who have never tested for HIV and those who have not tested within the previous 12 months | |

| **Supplementary Table 6a: Number of respondents with and without any chronic health conditions** | | | | | |
| --- | --- | --- | --- | --- | --- |
|  | **All number of consultations included**  **Unweighted N**  **(Weighted N)** | **Including those only with ≥1 consultations**  **Unweighted N**  **(Weighted N)** | **Including those only with ≥2 consultations**  **Unweighted N**  **(Weighted N)** | **Including those only with ≥3 consultations**  **Unweighted N**  **(Weighted N)** | **Including those only with ≥4 consultations**  **Unweighted N**  **(Weighted N)** |
| **Overall** | | | | | |
| Heterosexual men | 41,733  (12,432,427) | 27,247  (7,790,302) | 18,402  (5,110,372) | 11,900  (3,177,909) | 8,013  (2,047,636) |
| Heterosexual women | 49, 439  (12,784,406) | 37,756  (9,614,620) | 27,484  (6,806,817) | 18,759  (4,564,988) | 13,189  (3,165,172) |
| Gay and bisexual men | 1,157  (375,352) | 755  (240,084) | 550  (175,674) | 349  (105,573) | 246  (82,205) |
| Lesbian and bisexual women | 1,393  (414,591) | 1,057  (310,128) | 789  (228,462) | 586  (156,693) | 415  (108,644) |
|  | | | | | |
| **Among those with at least one chronic health condition** | | | | | |
| Heterosexual men | 22,008  (5,616,245) | 17, 254  (4,325,517) | 12,981  (3,211,753) | 9,049  (2,200,260) | 6,384  (1,493,015) |
| Heterosexual women | 26,982  (5,958,597) | 22,470  (4,957,019) | 17,662  (3,828,909) | 12,821  (2,757,745) | 9,335  (1,995,306) |
| Gay and bisexual men | 561  (145,835) | 428  (107,911) | 339  (88,802) | 230  (57,063) | 171  (45,634) |
| Lesbian and bisexual women | 675  (186, 607) | 560  (148,092) | 437  (112,963) | 348  (83,053) | 265  (63,010) |
|  | | | | | |
| **Among those with no chronic health conditions** | | | | | |
| Heterosexual men | 19,725  (6,816,183) | 9,993  (3,464,785) | 5,421  (1,898,619) | 2,851  (977,649) | 1,629  (554,621) |
| Heterosexual women | 22,457  (6,825,808) | 15,286  (4,657,601) | 9,822  (2,977,908) | 5,938  (1,807,243) | 3,854  (1,169,866) |
| Gay and bisexual men | 596  (229,517) | 327  (132,173) | 211  (86,872) | 119  (48,510) | 75  (36,571) |
| Lesbian and bisexual women | 718  (227,985) | 497  (162,035) | 352  (115,500) | 238  (73,640) | 150  (45,634) |
|  | | | | | |

| **Supplementary Table 6b: Comparing rate ratios by number of consultations with a primary care physician with and without chronic health conditions** | | | | | | |
| --- | --- | --- | --- | --- | --- | --- |
|  | **All number of consultations included**  **RR (95% CI)** | **All number of consultations including imputed values of HIV and number of sexual partners as covariates**  **RR (95% CI) ^a^** | **Including those only with ≥1 consultations**  **RR (95% CI) ^b^** | **Including those only with ≥2 consultations**  **RR (95% CI) ^b^** | **Including those only with ≥3 consultations**  **RR (95% CI) ^b^** | **Including those only with ≥4 consultations**  **RR (95% CI) ^b^** |
| **Overall** | | | | | | |
| Heterosexual men | Reference | Reference | Reference | Reference | Reference | Reference |
| Heterosexual women | 1.24 (1.20, 1.28) | 1.24 (1.20, 1.27) | 1.13 (1.09, 1.17) | 1.09 (1.05, 1.14) | 1.06 (1.01, 1.11) | 1.02 (0.97, 1.07) |
| Gay and bisexual men | 1.29 (1.12, 1.48) | 1.17 (1.02, 1.33) | 1.29 (1.13, 1.47) | 1.19 (1.03, 1.39) | 1.25 (1.07, 1.46) | 1.14 (0.96, 1.35) |
| Lesbian and bisexual women | 1.23 (1.10, 1.39) | 1.18 (1.07, 1.31) | 1.08 (0.96,1.22) | 1.04 (091, 1.19) | 1.01 (0.87, 1.17) | 0.99 (0.85, 1.17) |
|  | | | | | |  |
| **Among those with at least one chronic health condition** | | | | | | |
| Heterosexual men | Reference | Reference | Reference | Reference | Reference | Reference |
| Heterosexual women | 1.12 (1.07, 1.16) | 1.12 (1.08, 1.16) | 1.07 (1.03, 1.11) | 1.05 (1.00, 1.09) | 1.03 (0.98, 1.08) | 0.99 (0.94, 1.05) |
| Gay and bisexual men | 1.12 (0.97, 1.31) | 1.05 (0.91, 1.22) | 1.16 (1.00, 1.34) | 1.07 (0.90, 1.27) | 1.13 (0.93, 1.37) | 1.03 (0.82, 1.29) |
| Lesbian and bisexual women | 1.10 (0.91, 1.33) | 1.07 (0.91, 1.26) | 1.07 (0.88,1.29) | 1.08 (0.88, 1.32) | 1.08 (0.88, 1.33) | 1.04 (0.83, 1.30) |
|  | | | | | |  |
| **Among those with no chronic health conditions** | | | | | | |
| Heterosexual men | Reference | Reference | Reference | Reference | Reference | Reference |
| Heterosexual women | 1.46 (1.38, 1.54) | 1.46 (1.39, 1.54) | 1.27 (1.19, 1.35) | 1.22 (1.12, 1.31) | 1.15 (1.05, 1.25) | 1.08 (0.98, 1.07) |
| Gay and bisexual men | 1.49 (1.16, 1.91) | 1.30 (1.04, 1.63) | 1.47 (1.16, 1.86) | 1.39 (1.06, 1.84) | 1.44 (1.10, 1.88) | 1.33 (1.00, 1.75) |
| Lesbian and bisexual women | 1.41 (1.21, 1.65) | 1.34 (1.17, 1.53) | 1.11 (0.94, 1.30) | 1.00 (0.83, 1.20) | 0.91 (0.74, 1.12) | 0.93 (0.74, 1.18) |
| ^a^ HIV testing in the previous 12 months (yes/no) and reported number of sexual partners (continuous number) were multiply imputed using chained equations and 25 imputation sets and added as covariates. Estimates remained the same when comparing models that included just the HIV testing variable, and those in which both HIV testing and number of sexual partners were included. Here, we chose to showcase estimates when both imputed variables were included, to illustrate our approach in using them as proxies for sexual health clinics usage. Specifically, we hypothesized that individuals with more sexual partners would be more inclined to visit sexual health clinics, and that HIV tests are more likely to be conducted at these clinics.  ^b^ Truncated Poisson models were used  95% CIs derived using bootstrap sampling replicate weights provided in the CCHS | | | | | | |

Q. Are you taking a vitamin supplement containing folic acid? Yes/No, was asked only for women between the ages of 18-54 years. We employed this question as a proxy for potential pregnancy, influencing prenatal visits to a primary care physician, in light of Health Canada's recommendation for continuous folic acid supplement use during pregnancy [13]. Direct data on pregnancy was not available in the publicly available PUMF CCHS dataset.

| **Supplementary Table 7a: Estimates for respondents excluding women on folic acid supplements** | | | |
| --- | --- | --- | --- |
|  | **Overall**  **estimates**  **Unweighted N**  **(Weighted N)** | **Estimates for those with no chronic physical health conditions**  **Unweighted N**  **(Weighted N)** | **Estimates for those with ≥1 chronic health conditions**  **Unweighted N**  **(Weighted N)** |
| Heterosexual men | 41,733  (12,432,428) | 19,725  (6,816,183) | 22,008  (5,616,245) |
| Heterosexual women | 43,787  (10,975,863) | 18,606  (5,535,899) | 25,181  (5,439,964) |
| Gay and bisexual men | 1,157  (375,352) | 596  (229,517) | 561  (145,835) |
| Lesbian and bisexual women | 1,210  (365,066) | 620  (200,275) | 590  (164,790) |

| **Supplementary Table 7b: Rate ratios for the number of primary care physician consultations by sex and sexual identity, excluding women on folic acid supplements** | | | |
| --- | --- | --- | --- |
|  | **Overall**  **RR (95% CI)** | **No chronic physical health conditions**  **RR (95% CI)** | **≥1 chronic physical health conditions**  **RR (95% CI)** |
| Heterosexual men | Reference | Reference | Reference |
| Heterosexual women | 1.18 (1.14, 1.21) | 1.36 (1.28, 1.44) | 1.09 (1.05, 1.13) |
| Gay and bisexual men | 1.27 (1.11, 1.46) | 1.45 (1.13, 1.86) | 1.11 (0.95, 1.30) |
| Lesbian and bisexual women | 1.21 (1.06, 1.38) | 1.37 (1.16, 1.62) | 1.07 (0.86, 1.33) |
| All models were adjusted for the full set of covariates presented in Table 3 of the main manuscript.  RRs are not significantly different between heterosexual women, gay or bisexual men, and lesbian or bisexual women. | | | |

| **Supplementary Table 8: Profile of heterosexual women, gay & bisexual men, and lesbian & bisexual women across predisposing, enabling and need-based factors across consultation numbers** | | | | | | |
| --- | --- | --- | --- | --- | --- | --- |
|  | **No consultations**  **N = 12,421**  **(weighted N = 3,409,516)** | | | **1 consultation**  **N = 10,745**  **(weighted N = 2,953,879)** | | |
|  | Heterosexual women  N = 11,683  (wt. N = 3,169,785) | Gay & bisexual men  N = 402  (wt. N = 135,268) | Lesbian & bisexual women  N=336  (wt. N = 104,464) | Heterosexual women  N = 10,272  (wt. N = 2,807,803) | Gay & bisexual men  N=205  (wt. N = 64,410) | Lesbian & bisexual women  N=268  (wt. N = 81,666) |
|  | Weighted %  (95% CI) | Weighted %  (95% CI) | Weighted %  (95% CI) | Weighted %  (95% CI) | Weighted %  (95% CI) | Weighted %  (95% CI) |
| **Age (years)**  18 – 29  30 – 39  40 – 49  50 – 59  60 – 69  ≥70 | 24.8 (23.4, 26.2)  18.9 (17.7, 20.1)  18.2 (17.0, 19.5)  16.3 (15.3, 17.3)  12.6 (11.7, 13.5)  9.2 (8.6, 9.9) | 38.6 (29.4, 48.7)  19.2 (13.3, 26.9)  15.0 (9.7, 22.5)  13.2 (9.3, 18.2)  9.9 (4.3, 21.6)  4.0 (1.9, 8.0) | 45.3 (34.8, 56.2)  19.5 (11.6, 30.8)  10.4 (5.7, 18.2)  11.4 (6.7, 18.8)  11.4 (3.3, 32.5)  2.0 (0.9, 3.9) | 16.5 (15.2, 17.8)  16.5 (15.3, 17.7)  18.9 (17.7, 20.3)  20.4 (19.2, 21.6)  16.9 (15.8, 18.1)  10.8 (10.1, 11.6) | 25.2 (17.0, 35.6)  22.0 (12.4, 36.1)  15.0 (8.6, 24.7)  19.9 (13.4, 28.7)  11.4 (7.3, 17.2)  6.6 (3.8, 11.2) | 39.9 (31.2, 49.4)  20.5 (13.3, 30.3)  9.3 (5.1, 16.3)  15.5 (10.5, 22.4)  10.5 (6.5, 16.7)  4.2 (2.3, 7.3) |
| **<Secondary education** | 11.8 (10.9, 12.6) | 5.1 (3.2, 8.0) | 20.7 (10.4, 36.8) | 9.2 (8.3, 10.0) | 5.5 (2.6, 11.3) | 9.8 (5.8, 16.0) |
| **Student** | 13.4 (12.2, 14.7) | 13.9 (8.1, 22.9) | 23.7 (15.0, 35.4) | 9.7 (8.7, 10.8) | 17.8 (9.6, 30.4) | 18.4 (12.3, 26.7) |
| **Racialized** | 25.5 (23.9, 27.3) | 18.5 (12.4, 26.7) | 17.4 (10.9, 26.7) | 16.5 (14.9, 18.1) | 7.7 (3.3, 17.0) | 11.8 (6.1, 21.6) |
| **Immigrant** | 28.0 (26.4, 29.7) | 20.2 (14.0, 28.3) | 9.9 (5.4, 17.5) | 21.8 (20.2, 23.4) | 17.7 (8.8, 32.3) | 14.4 (8.3, 23.9) |
| **Single** | 27.8 (26.3, 29.3) | 65.2 (56.6, 72.8) | 64.3 (53.7, 73.7) | 19.7 (18.4, 21.1) | 44.3 (33.9, 55.2) | 53.3 (44.2, 62.3) |
| **Very strong sense of belonging to local community** | 17.4 (16.2, 18.6) | 13.4 (8.8, 19.9) | 9.3 (5.4, 15.6) | 17.4 (16.2, 18.8) | 16.4 (9.6, 25.6) | 15.6 (9.5, 24.6) |
| **Annual household income < $40,000** | 26.2 (24.8, 27.7) | 23.1 (17.5, 29.8) | 35.8 (25.1, 48.2) | 19.5 (18.4, 20.8) | 17.2 (11.8, 24.5) | 25.9 (19.2, 34.0) |
| **Has a regular health care provider** | 72.1 (70.7, 73.4) | 62.0 (53.1, 70.2) | 48.7 (37.8, 59.7) | 90.7 (89.7, 91.6) | 80.5 (71.6, 87.2) | 88.5 (82.1, 92.8) |
| **Has some insurance for medication cost** | 76.3 (74.8, 77.7) | 64.0 (52.9, 73.7) | 69.4 (58.2, 78.6) | 82.3 (80.9, 83.5) | 81.0 (73.1, 87.1) | 78.5 (70.5, 84.8) |
| **Living alone** | 15.2 (14.1, 16.4) | 27.2 (20.9, 34.5) | 16.9 (12.2, 22.9) | 15.1 (13.9, 16.2) | 23.4 (16.9, 31.4) | 23.2 (16.7, 31.3) |
| **Fair or poor self-perceived general health** | 6.4 (5.7, 7.2) | 7.5 (4.8, 11.6) | 11.8 (3.7, 31.8) | 5.0 (4.4, 5.6) | 5.1 (2.7, 9.3) | 5.1 (2.9, 8.7) |
| **Quite a bit or extremely self-perceived life stress** | 20.5 (19.3, 21.8) | 25.7 (18.4, 34.8) | 31.1 (21.7, 42.4) | 22.0 (20.7, 23.5) | 25.9 (15.6, 39.9) | 28.7 (21.0, 37.9) |
| **Fair or poor self-perceived mental health** | 3.7 (3.2, 4.3) | 7.9 (4.9, 12.6) | 12.2 (5.5, 24.9) | 3.6 (3.0, 4.2) | 10.8 (5.0, 21.9) | 15.8 (10.6, 22.9) |
| **Chronic physical health conditions**  None  One  Two  ≥Three | 68.4 (67.0, 69.7)  18.8 (17.7, 19.9)  7.4 (6.7, 8.2)  5.4 (4.9, 5.9) | 71.9 (62.1, 80.1)  20.9 (13.3, 31.2)  4.6 (2.4, 8.9)  2.5 (1.5, 4.0) | 63.1 (50.4, 74.3)  18.7 (12.9, 26.2)  6.6 (3.5, 11.8)  11.6 (3.3, 33.6) | 59.8 (58.3, 61.4)  24.0 (22.6, 25.3)  10.2 (9.3, 11.2)  6.0 (5.3, 6.8) | 70.3 (60.9, 78.3)  20.0 (13.7, 28.3)  5.3 (2.9, 9.8)  4.2 (2.2, 7.9) | 56.9 (47.4, 66.1)  25.2 (17.9, 34.1)  7.9 (4.8, 12.6)  9.9 (5.3, 17.9) |
| **Mood or anxiety disorder** | 7.6 (6.8, 8.4) | 12.7 (7.9, 19.7) | 22.6 (14.2, 33.8) | 9.9 (9.0, 10.9) | 15.7 (8.9, 26.2) | 30.3 (22.0, 39.9) |
| **Heavy drinker** | 16.3 (15.1, 17.5) | 32.8 (24.2, 42.8) | 20.9 (14.6, 28.9) | 16.1 (14.9, 17.4) | 25.5 (17.2, 36.1) | 23.5 (16.9, 31.7) |
| **Mean number of emergency room visits in the previous 12 months** | 0.33 (0.31, 0.36) | 0.24 (0.12, 0.35) | 0.54 (0.30, 0.78) | 0.28 (0.26, 0.31) | 0.41 (0.21, 0.61) | 0.35 (0.23, 0.48) |

| **Supplementary Table 8 (continued): Profile of heterosexual women, gay & bisexual men, and lesbian & bisexual women across predisposing, enabling and need-based factors across consultation numbers** | | | | | | |
| --- | --- | --- | --- | --- | --- | --- |
|  | **2-3 consultations**  **N = 14,973**  **(weighted N = 3,854,932)** | | | **≥4 consultations**  **N = 13,850**  **(weighted N = 3,356,021)** | | |
|  | Heterosexual women  N = 14,295  (wt. N = 3,641,645) | Gay & bisexual men  N = 304  (wt. N = 93,469) | Lesbian & bisexual women  N=374  (wt. N = 119,818) | Heterosexual women  N = 13,189  (wt. N = 3,165,172) | Gay & bisexual men  N=246  (wt. N = 82,205) | Lesbian & bisexual women  N=415  (wt. N = 108,644) |
|  | Weighted %  (95% CI) | Weighted %  (95% CI) | Weighted %  (95% CI) | Weighted %  (95% CI) | Weighted %  (95% CI) | Weighted %  (95% CI) |
| **Age (years)**  18 – 29  30 – 39  40 – 49  50 – 59  60 – 69  ≥70 | 16.9 (15.8, 18.1)  17.1 (16.1, 18.2)  16.5 (15.5, 17.6)  19.2 (18.2, 20.4)  16.7 (15.7, 17.7)  13.5 (12.9, 14.2) | 29.9 (20.7, 41.1)  14.5 (9.7, 21.1)  14.3 (8.7, 22.6)  21.5 (13.7, 31.9)  12.6 (8.1, 19.1)  7.1 (4.7, 10.7) | 41.1 (32.2, 50.4)  19.9 (14.6, 26.7)  14.7 (8.8, 23.3)  12.8 (8.4, 19.0)  8.8 (4.6, 16.2)  2.6 (1.5, 4.5) | 15.2 (14.2, 16.3)  18.2 (17.1, 19.4)  15.7 (14.6, 16.9)  18.3 (17.2, 19.4)  16.7 (15.7, 17.7)  16.0 (15.2, 16.9) | 24.9 (16.8, 35.5)  11.4 (7.3, 17.4)  14.4 (8.3, 23.9)  23.3 (15.6, 33.4)  12.5 (8.3, 18.6)  13.4 (7.0, 24.0) | 44.4 (37.1, 52.1)  20.4 (15.6, 26.3)  13.7 (9.3, 19.7)  11.2 (7.7, 16.1)  6.8 (4.5, 10.1)  3.3 (2.1, 5.4) |
| **<Secondary education** | 10.7 (9.9, 11.5) | 6.3 (3.3, 11.7) | 10.7 (6.9, 16.2) | 13.7 (12.8, 14.7) | 5.1 (2.9, 8.8) | 10.8 (7.3, 15.7) |
| **Student** | 9.6 (8.7, 10.6) | 16.8 (10.1, 26.5) | 21.8 (14.3, 31.7) | 8.1 (7.3, 9.1) | 7.6 (3.8, 14.4) | 18.9 (13.3, 26.1) |
| **Racialized** | 19.9 (18.6, 21.4) | 21.1 (13.3, 31.9) | 17.7 (10.7, 27.9) | 22.6 (21.2, 24.1) | 20.5 (12.5, 31.8) | 11.9 (7.0, 19.3) |
| **Immigrant** | 24.8 (23.4, 26.2) | 18.0 (11.7, 26.9) | 16.8 (9.6, 27.7) | 27.5 (26.2, 29.0) | 26.1 (17.6, 36.7) | 16.6 (10.9, 24.4) |
| **Single** | 20.1 (18.9, 21.4) | 49.2 (39.5, 58.9) | 42.3 (33.8, 51.3) | 18.8 (17.6, 20.0) | 54.4 (44.3, 64.2) | 46.2 (38.6, 54.0) |
| **Very strong sense of belonging to local community** | 18.0 (17.0, 19.1) | 18.8 (11.7, 29.7) | 8.7 (5.4, 13.9) | 19.5 (18.4, 20.7) | 13.6 (8.5, 21.1) | 7.2 (4.8, 10.6) |
| **Annual household income < $40,000** | 21.7 (20.6, 22.9) | 24.8 (17.5, 33.8) | 25.6 (19.3, 33.0) | 27.2 (25.9, 28.5) | 27.0 (18.8, 37.3) | 33.1 (26.6, 40.4) |
| **Has a regular health care provider** | 93.0 (92.2, 93.7) | 88.9 (82.6, 93.2) | 87.2 (81.0, 91.5) | 94.7 (94.0, 95.2) | 87.6 (77.5, 93.6) | 90.6 (84.9, 94.3) |
| **Has some insurance for medication cost** | 81.7 (80.5, 82.8) | 84.2 (77.5, 89.1) | 81.9 (75.3, 87.0) | 81.3 (80.2, 82.4) | 87.4 (80.9, 91.9) | 77.4 (70.1, 83.2) |
| **Living alone** | 17.2 (15.9, 18.4) | 31.6 (24.0, 40.3) | 15.0 (10.2, 21.6) | 19.2 (17.9, 20.6) | 33.9 (25.0, 44.0) | 19.7 (15.4, 24.9) |
| **Fair or poor self-perceived general health** | 9.3 (8.5, 10.1) | 5.8 (3.4, 9.6) | 9.7 (6.4, 14.4) | 21.9 (20.7, 23.0) | 24.9 (17.3, 34.7) | 29.8 (23.3, 37.4) |
| **Quite a bit or extremely high self-perceived life stress** | 23.3 (22.0, 24.5) | 25.0 (17.8, 33.8) | 27.8 (20.8, 36.1) | 27.4 (26.1, 28.8) | 28.4 (20.5, 37.9) | 42.9 (35.7, 50.4) |
| **Fair or poor self-perceived mental health** | 6.0 (5.3, 6.7) | 9.2 (5.7, 14.6) | 16.0 (11.2, 22.4) | 13.3 (12.3, 14.4) | 22.8 (15.3, 32.5) | 36.7 (29.5, 44.6) |
| **Chronic physical health conditions**  None  One  Two  ≥Three | 49.6 (48.3, 51.1)  26.5 (25.3, 27.7)  13.2 (12.3, 14.2)  10.6 (10.0, 11.3) | 53.8 (44.1, 63.3)  27.8 (19.6, 37.9)  11.7 (7.7, 17.5)  6.6 (4.2, 10.2) | 58.3 (49.2, 66.8)  18.4 (12.5, 26.4)  12.2 (7.1, 20.2)  11.1 (7.4, 16.3) | 37.0 (35.6, 38.4)  24.7 (23.5, 25.9)  16.7 (15.7, 17.8)  21.6 (20.5, 22.8) | 44.5 (34.4, 55.1)  18.6 (12.8, 26.2)  17.7 (11.3, 26.6)  19.3 (12.8, 27.9) | 42.0 (34.6, 49.8)  22.5 (17.1, 29.0)  17.3 (12.4, 23.6)  18.2 (13.0, 24.9) |
| **Mood or anxiety disorder** | 14.5 (13.6, 15.4) | 18.7 (13.2, 25.9) | 42.2(33.8, 51.1) | 27.1 (25.8, 28.4) | 35.4 (26.7, 45.2) | 61.5 (54.0, 68.4) |
| **Heavy drinker** | 16.1 (15.1, 17.2) | 36.7 (26.9, 47.5) | 36.5 (27.8, 46.3) | 13.5 (12.5, 14.5) | 26.2 (17.8, 36.7) | 24.9 (19.0, 31.9) |
| **Mean number of emergency room visits in the previous 12 months** | 0.42 (0.40, 0.45) | 0.29 (0.20, 0.39) | 0.59 (0.40, 0.78) | 0.83 (0.78, 0.88) | 0.63 (0.42, 0.84) | 1.59 (1.12, 2.07) |

**References**

1. **Regularization Part 2: Lasso (L1) regression** [https://www.youtube.com/watch?v=NGf0voTMlcs&t=53s]

2. **Stata Lasso Reference Manual Release 18** [https://www.stata.com/manuals/lasso.pdf]

3. Hastie T, Tibshirani R, Wainwright M: **Statistical learning with sparsity : the lasso and generalizations**. In*.*, 1st. edn. Boca Raton: Chapman & Hall/CRC; 2015: 7-40.

4. Hossain S, Ahmed E: **Shrinkage and Penalty Estimators of a Poisson Regression Model**. *Australian & New Zealand Journal of Statistics* 2012, **54**(3):359-373.

5. **Chapter 19: Adaptive Lasso** [https://yaydede.github.io/toolbox/adaptive-lasso.html]

6. Zou H: **The Adaptive Lasso and Its Oracle Properties**. *Journal of the American Statistical Association* 2006, **101**(476):1418-1429.

7. **A plug-in for Poisson lasso and a comparison of partialing-out Poisson estimators that use different methods for selecting the lasso tuning parameters** [https://www.econ.queensu.ca/sites/econ.queensu.ca/files/cspplugin.pdf]

8. Belloni A, Chernozhukov V: **High Dimensional Sparse Econometric Models: An Introduction**. In: *Inverse Problems and HIgh-Dimensional Estimation, Lecture Notes in Statistics. Volume 203*, edn.: Springer; 2011: 121-156.

9. **Using Double-Lasso Regression for Principled Variable Selection** [https://home.uchicago.edu/ourminsky/Variable_Selection.pdf]

10. Belloni A, Chernozhukov V, Hansen C: **Inference on Treatment Effects after Selection among High-Dimensional Controls**. *The Review of Economic Studies* 2013, **81**(2):608-650.

11. Belloni A, Chernozhukov V, Hansen C: **High-Dimensional Methods and Inference on Structural and Treatment Effects**. *Journal of Economic Perspectives* 2014, **28**(2):29-50.

12. **Mostly Dangerous Econometrics: How to do Model Selection with Inference in Mind** [https://stuff.mit.edu/~vchern/papers/Chernozhukov-Saloniki.pdf]

13. **Prenatal Nutritional Guidelines for Health Professionals: Folate** [https://www.canada.ca/content/dam/hc-sc/migration/hc-sc/fn-an/alt_formats/hpfb-dgpsa/pdf/pubs/folate-eng.pdf]
